# TYROBP/DAP12 knockout in Huntington’s disease Q175 mice cell-autonomously decreases microglial expression of disease-associated genes and non-cell-autonomously mitigates astrogliosis and motor deterioration

**DOI:** 10.1186/s12974-024-03052-4

**Published:** 2024-03-08

**Authors:** Jordi Creus-Muncunill, Jean Vianney Haure-Mirande, Daniele Mattei, Joanna Bons, Angie V. Ramirez, B. Wade Hamilton, Chuhyon Corwin, Sarah Chowdhury, Birgit Schilling, Lisa M. Ellerby, Michelle E. Ehrlich

**Affiliations:** 1 Department of Neurology, Icahn School of Medicine at Mount Sinai, New York, USA; 2 Nash Family Department of Neuroscience and Friedman Brain Institute, Icahn School of Medicine at Mount Sinai, New York, USA; 3 Buck Institute for Research on Aging, Novato, CA USA

**Keywords:** Complement, Huntington’s disease, Microglia, Multi-omics, Neuroinflammation, Q175, TYROBP

## Abstract

**Introduction:**

Huntington’s disease (HD) is a fatal neurodegenerative disorder caused by an expansion of the CAG trinucleotide repeat in the Huntingtin gene (*HTT*). Immune activation is abundant in the striatum of HD patients. Detection of active microglia at presymptomatic stages suggests that microgliosis is a key early driver of neuronal dysfunction and degeneration. Recent studies showed that deletion of *Tyrobp*, a microglial protein, ameliorates neuronal dysfunction in Alzheimer’s disease amyloidopathy and tauopathy mouse models while decreasing components of the complement subnetwork.

**Objective:**

While TYROBP/DAP12-mediated microglial activation is detrimental for some diseases such as peripheral nerve injury, it is beneficial for other diseases. We sought to determine whether the TYROBP network is implicated in HD and whether *Tyrobp* deletion impacts HD striatal function and transcriptomics.

**Methods:**

To test the hypothesis that *Tyrobp* deficiency would be beneficial in an HD model, we placed the Q175 HD mouse model on a *Tyrobp*-null background. We characterized these mice with a combination of behavioral testing, immunohistochemistry, transcriptomic and proteomic profiling. Further, we evaluated the gene signature in isolated Q175 striatal microglia, with and without *Tyrobp.*

**Results:**

Comprehensive analysis of publicly available human HD transcriptomic data revealed that the TYROBP network is overactivated in the HD putamen. The Q175 mice showed morphologic microglial activation, reduced levels of post-synaptic density-95 protein and motor deficits at 6 and 9 months of age, all of which were ameliorated on the *Tyrobp*-null background. Gene expression analysis revealed that lack of *Tyrobp* in the Q175 model does not prevent the decrease in the expression of striatal neuronal genes but reduces pro-inflammatory pathways that are specifically active in HD human brain, including genes identified as detrimental in neurodegenerative diseases, e.g. *C1q* and members of the *Ccr5* signaling pathway. Integration of transcriptomic and proteomic data revealed that astrogliosis and complement system pathway were reduced after *Tyrobp* deletion, which was further validated by immunofluorescence analysis.

**Conclusions:**

Our data provide molecular and functional support demonstrating that *Tyrobp* deletion prevents many of the abnormalities in the HD Q175 mouse model, suggesting that the *Tyrobp* pathway is a potential therapeutic candidate for Huntington’s disease.

**Supplementary Information:**

The online version contains supplementary material available at 10.1186/s12974-024-03052-4.

## Background

Huntington’s disease (HD) is a neurodegenerative disease caused by an expansion of the trinucleotide CAG within exon-1 of the Huntingtin (*HTT*) gene. The resultant protein (mutant Huntingtin; mHtt) contains an aberrant polyglutamine tail to which neurotoxicity is attributed [1]. HD is characterized by a progressive loss of striatal projection neurons, known as medium spiny neurons (MSNs) [2, 3], which leads to motor alterations, cognitive deficits, and eventual death. Neurodegeneration, however, is not restricted to the striatum since cortical atrophy is also evident in HD patients [4, 5]. There are several cell-autonomous and non-cell-autonomous mechanisms by which the *HTT* mutation causes disease [6], including a role for glial cells in both the onset and progression of the disease in neurons [7]. mHtt aggregates accumulate in microglia [8] and brains from presymptomatic HD human carriers contain activated microglia, which have been identified as morphologically abnormal, with increased size, amoeboid-like cell body shape, short or absent processes, and increased phagocytic activity [9–11]. Accordingly, there is also an increase of pro-inflammatory cytokines, including IL-1β, in HD individuals [12–14]. Transcriptional profiling of HD brains revealed that pro-inflammatory pathways are activated in the most affected brain regions, i.e. caudate/putamen and cortex [15–17]. HD mouse models largely fail to recapitulate the activation of pro-inflammatory pathways at a transcriptional level [18], but the question as to whether restriction of mHtt expression in microglia is sufficient to cause disease is unresolved [19, 20]. Notably, depletion of microglia in the R6/2 mouse HD model alleviates many of the components of the HD phenotype, including astrogliosis, motor deficits, and volume loss [21].

Although microglia have been reported to be abnormal in HD, they do not obviously express the Alzheimer’s disease (AD) Disease Associated Microglia (DAM) phenotype [22–24]. The DAM phenotype is characterized by multiple microglia transcriptomic subtypes with the involvement of a group of genes, but overall, there is a decrease in the expression of homeostatic markers and an increase in many genes involved in neuroinflammation, including complement factors, C1X, TREM2 and its obligate adaptor, TYROBP, APOE, SPP1, and members of the CCL gene family. In the brain, TYROBP is the adaptor for disease associated proteins  other than TREM2, including CR3. It is a microglial phosphotyrosine phosphoprotein that has been genetically associated with late onset Alzheimer’s disease (LOAD) [24]. Mechanistically, TYROBP works in protein complexes to activate SYK, Akt, and Erk, and to regulate phagocytosis and signal transduction [25–27]. With the knowledge that *Tyrobp* deletion leads to a decrease in C1Q in the presence of either an amyloidopathy or a tauopathy, we asked whether this would also occur in the presence of the *Htt* proteinopathy, along with alterations in other DAM genes impacted by TYROBP which are contained in its network [28]. Using a computational approach of published databases, *TYROBP* was identified as a hub gene and node in a complement pathway driving neurodegeneration in AD. In this seminal publication, the *TYROBP* network was specifically noted to be undetectable in HD, which was used as a “negative” control [24]. In our previous studies, deletion of *Tyrobp* (also known as DAP12) in AD amyloidopathy and tauopathy mouse models mitigates cognitive dysfunction and restores synaptic function, while decreasing microglial clustering around amyloid plaques [28–30]. On a molecular level, deletion of *Tyrobp* in a model of amyloidopathy reversed the complement related network contained within the disease-associated microglia gene expression changes, including a decrease in C1Q. In a tauopathy model in which DAM genes are not identified, deletion of *Tyrobp* also decreases the level of C1Q from homeostatic levels [28, 29].

We reassessed the status of a *Tyrobp* network in HD using additional RNA sequencing databases that have become available since the original publication, and found it to be enriched. Thus, with the knowledge that the clinical effects of decreasing C1Q activity in human HD is under active investigation with ANX005, an antibody targeting C1Q, and with the increasing characterization of the role of microglia in HD pathogenesis, we sought to investigate the impact of deletion of *Tyrobp* in the HD Q175 mouse model. The microglial DEGs noted in the bulk RNA-seq were again identified, as were other highly relevant DEGs, including *Spp1* and *Ccl4*.

## Methods

### Data sources and wikipathway enrichment analysis

To identify the pathways enriched for upregulated genes in HD human brains, we used publicly available HD human transcriptomic data providing differentially expressed genes or pathway enrichment analysis. We filtered all datasets on gene expression omnibus (GEO) or European Nucleotide Archive (ENA) databases based on the organism and considering both RNA-seq and microarray sequencing methods. The query was limited to all datasets relevant to HD human brain research and high throughput genomics experiments which would include up to 6 entries. PRJEB44140 (RNA-seq, striatum [31]), GSE64810 (RNA-seq, Brodmann Area 9, [32]), Al-Dalahmah et al. [33] (RNA-seq, anterior cingulate cortex, GSE26927 (microarray, caudate, [16]), GSE3790 (microarray, caudate, Broadmann area 4 and cerebellum, [34]) were analyzed. Pathway enrichment analysis was performed using the EnrichR package [35] considering only upregulated differentially expressed protein-coding genes (Fold Change > 0.5 and adjusted p-value < 0.05). Significant pathways were identified by Fisher’s exact test with adjusted p-value < 0.05. All gene sets provided a list of differentially expressed genes except GSE26927. For GSE26927, we used GEO2R online platform to retrieve DEGs. Data were log normalized, and the Benjamini and Hochberg False Discovery Rate (FDR) method was used to adjust the p-values. GSE79666 dataset, although it contains RNA-seq data of the BA4 motor cortex of 7 control and 7 Huntington's disease patients, was excluded because only 51 DEGs were detected. For GSE3790, which contains microarray data from the caudate, cortex and cerebellum, the authors do not provide adjusted p-values. We set up the threshold as nominal p-value < 0.001, as suggested by the authors.

### Network construction of protein–protein interaction

To inquire into the role of common upregulated DEGs across HD brains, we analyzed these genes by means of STRING v11.5 tool. The minimum required interaction score was set to high confidence (0.7). Text mining, experiments, databases, co-expression, neighborhood, gene fusion and co-occurrence were used as prediction methods. The plug-in Molecular Complex Detection (MCODE), a well-known automated method to find highly interconnected subgraphs as molecular complexes or clusters in large PPI networks, was used to screen the modules or clusters of PPI network in Cytoscape.

### Mice

Animal procedures were conducted in accordance with the NIH Guidelines for the Care and Use of Experimental Animals and were approved by the Institutional Animal Care and Use Committee at Icahn School of Medicine at Mount Sinai. All mice were on a C57Bl6/J background. Heterozygote Q175 (JAX #029928) and *Tyrobp* knockout (*Tyrobp*^(−/−)^) [36] mice were obtained from Jackson Laboratories and Taconic/Merck Laboratory, respectively. Mice were kept in a 12-h light–dark cycle with ad libitum access to food and water at Icahn School of Medicine at Mount Sinai, NY, USA.

### Tissue extraction

10-month-old mice were anesthetized with pentobarbital (50 mg/kg intraperitoneal) and perfused with ice-cold phosphate-buffered saline (PBS). Brains were removed, hemispheres were sagittally separated, and the striatum was dissected from the left hemisphere and flash frozen. The right hemisphere and hindbrain were post-fixed in 4% paraformaldehyde for 48 h and prepared for histological analyses. The frozen striatum was used for RNA extraction and RT-qPCR as well as western blot analysis.

### Immunohistochemistry

30 μm sagittal free-floating sections were washed with 1X Tris Buffered Saline (TBS), blocked at room temperature (RT) for 1 h (5% goat serum, 0.25% Triton X-100, 1X TBS), and then incubated at 4ºC overnight with mouse anti-DARPP-32 (1:2000, sc-271111, Santa Cruz), rabbit anti-Iba1 (1:500, 019-1974, Wako), rat anti-CD68 (1:500, MCA1957, Bio-Rad) or mouse anti-PSD-95 (1:500, MAB1596, Millipore) antibodies in 1X TBS, 0.05% Triton X-100 with 1% goat serum. Sections were washed with 1X TBS + 0.1% Triton X-100 and incubated with the appropriate secondary antibody: anti-rabbit Alexa 488 (1:400, #A-11034, Thermo Fisher Scientific) or anti-rat Alexa 594 (1:500, #A-11007, Thermo Fisher Scientific). Images were obtained with Zeiss 700 confocal microscope (Zeiss, Thornwood, USA). Morphological analysis on microglia was performed as described in [37]. Intensity of fluorescence was calculated using the “measure” function in Fiji (v2.0.0.0). The freehand region of interest (ROI) tool was used to outline a specific area, equal in all images, and included an area of “no fluorescence” which was considered the background. The program then calculated the mean fluorescence. Microglia and CD68 were quantified using the same program with ROIs imaged by manual tracing, and counting of particles. For PSD-95 quantitation, three independent coronal brain sections were used for each mouse, containing the dorsal striatum. The 5 μm-thick confocal scans (optical section depth 0.33 mm, 15 sections per scan, imaged area per scan, ¼ of 20.945 mm^2^) of the synaptic zone (thereby excluding perinuclear puncta) in dorsal striatum were performed at 63X magnification on a Leica SP5 confocal laser-scanning microscope.

### RNA extraction and qPCR analysis

RNAs were isolated from mouse striata using the QIAzol® Lysis Reagent (Qiagen) and the miRNeasy® Micro Kit (Qiagen). 500 ng of total RNAs were reverse transcribed using the High-Capacity RNA-to-cDNA Kit (ThermoFisher, #4387406). cDNAs were subjected to real-time qPCR in a Step­One Plus system (Applied Biosystem) using The All-in-One qPCR Mix (GeneCopoeia, #QP001-01). Sequences of oligonucleotides used are: Mm99999915_g1_Gapdh (4331182, ThermoFisher),

C1q: C1q: Fwd 5′-AACCTCGGATACCAGTCCG-3′; Rev 5′-ATGGGGCTCCAGGAAATC-3′

CD68: Fwd 5′-TGTCTGATCTTGCTAGGACCG-3′; Rev 5′-GAGAGTAACGGCCTTTTTGTGA-3′

The individual value for each test transcript, performed in triplicate, was normalized to the abundance of GAPDH. Relative quantification was performed using the ΔΔCt method [38] and was expressed as fold-change relative to control by calculating 2-ΔΔCt.

### RNA-seq

Bulk RNA seq was performed by Novogene (https://en.novogene.com) using Illumina Novaseq 6000 S4 flow cells. Microglia RNA-seq was performed by Genomics Technology Facility, Department of Genetics and Genomic Sciences, Icahn School of Medicine at Mount Sinai using SMART-Seq v4 Ultra Low Input. Samples with RNA integrity number (RIN) > 8 were used. Non-directional libraries were constructed with a NEB kit using the manufacturer’s protocol. RNA-seq assays were performed after ribosomal RNA depletion by Ribo-Zero. FASTQ files were aligned to the annotated *Mus musculus* reference genome version GRCm38 using STAR (v2.5). FeatureCounts was used to quantify gene expression at the gene level based on UCSC gene model. Genes with at least 1 count per million in at least one sample were considered expressed and retained for further analysis. Differential expression analysis between two conditions/group was performed using the DESeq2 R package (1.14.1). The resulting p-values were adjusted using Benjamini and Hochberg's approach for controlling the FDR.

### KEGG pathway and reactome pathway analysis

GO terms, Bioplanet, and KEGG pathway analysis of differentially expressed genes were performed using Enrichr tool [35]. Pathways with p-value < 0.05 were considered significantly enriched by differential expressed genes if they had a minimum of 3 genes associated.

### Gene set enrichment analysis

In order to understand the biology underlying the gene expression profile, we performed pathway analysis using GSEA. We used GSEA because it is a threshold-free method that can detect pathway changes more sensitively and robustly than some methods [39]. The following options were selected or input into the software for analysis: gene set database:—Canonical pathways: c2.cp.v72.symbols.gmt; GO terms: c5.go.v7.2.symbols.gmt; KEGG pathways: c2.cp.kegg.v7.4.symbols.gmt. Number of permutations: 1,000. Permutation type: gene_set. Chip platform: Mouse_ENSEMBL_Gene_ID_Human_Orthologs_ MSigDB.v7.2.chip.

### Proteomics analysis

#### Proteolytic digestion and desalting

The striatum was dissected from the brains of 4 different mouse strains with 5 or 6 replicates each: strains used and compared were wild-type (WT, n = 5), *Tyrobp*^(−/−)^ (n = 5), Q175 (n = 6) and Q175;*Tyrobp*^(−/−)^ (n = 6). Frozen mouse striatum was subjected to 400 μL of lysis buffer composed of 8 M urea, 2% SDS, 200 mM triethylammonium bicarbonate (TEAB), pH 8.5, 75 mM NaCl, 1 μM trichostatin A, 3 mM nicotinamide, and 1 × protease/phosphatase inhibitor cocktail, and homogenized for 1 cycle with a Bead Beater TissueLyser II (QIAGEN, Germantown, MD) at 25 Hz for 1.5 min. Lysates were clarified by spinning at 15,700 × *g* for 15 min at 4 °C, and the supernatant containing the soluble proteins was collected. Protein concentrations were determined using a Bicinchoninic Acid Protein (BCA) Assay (Thermo Fisher Scientific, Waltham, MA), and subsequently 100 μg of protein from each sample were aliquoted and samples were brought to an equal volume using water. Samples were then solubilized using 4% SDS, 50 mM TEAB at a pH ~ 7.55. Proteins were reduced using 20 mM DTT in 50 mM TEAB for 10 min at 50 °C followed by 10 min at RT, and proteins were subsequently alkylated using 40 mM iodoacetamide in 50 mM TEAB for 30 min at RT in the dark. Samples were acidified with 12% phosphoric acid to obtain a final concentration of 1.2% phosphoric acid, and diluted with seven volumes of S-Trap buffer (90% methanol in 100 mM TEAB, pH ~ 7). Samples were then loaded onto the S-Trap micro spin columns (Protifi, Farmingdale, NY), and spun at 4000 × *g* for 10 s. The S-Trap columns were washed with S-Trap buffer twice at 4000 × *g* for 10 s each, before adding a solution of Sequencing-Grade Endoproteinase Lys-C (Sigma, Atlanta, GA) in 50 mM TEAB at a 1:20 (w/w) enzyme:protein ratio at 37 °C for 2 h. A solution of sequencing grade trypsin (Promega, San Luis Obispo, CA) in 50 mM TEAB at a 1:25 (w/w) enzyme:protein ratio was then added, and after a 1-h incubation at 47 °C, trypsin solution was added again at the same ratio, and proteins were digested overnight at 37 °C. Peptides were sequentially eluted with 50 mM TEAB (spinning for 1 min at 1000 × *g*), 0.5% formic acid (FA) in water (spinning for 1 min at 1000 × *g*), and 50% acetonitrile (ACN) in 0.5% FA (spinning for 1 min at 4000 × *g*). After vacuum drying, samples were resuspended in 0.2% FA in water, desalted with Oasis 10-mg Sorbent Cartridges (Waters, Milford, MA). All samples were vacuum dried and resuspended in 0.2% FA in water at a final concentration of 1 μg/μL. Finally, indexed retention time standard peptides (iRT; Biognosys, Schlieren, Switzerland) [40] were spiked in the samples according to manufacturer’s instructions.

#### Generation of the spectral library

For generating the spectral library, 15 μL from each digested striatum sample were pooled, and subsequently, an aliquot of 100 μg digested protein (from the pooled mouse striatum) was vacuum dried, resuspended in 300 μL of 0.1% trifluoroacetic acid (TFA), and fractionated using the Pierce High-pH Reversed-Phase Peptide Fractionation (HPRP) Kit (Thermo Fisher Scientific, Rockford, IL) according to manufacturer’s instructions. Eight fractions were eluted using 5%, 7.5%, 10%, 12.5%, 15%, 17.5%, 20% and 50% ACN in 0.1% triethylamine, then the 8 collected fractions were vacuum dried, and each fraction was resuspended in 12.5 μL of 0.2% FA. iRT peptides were spiked in the samples according to manufacturer’s instructions. To build the spectral library each fraction was acquired by DIA by LC–MS/MS (see below). Additionally, triplicate DIA measurements of the unfractionated pooled samples were performed.

#### Mass spectrometric analysis: data-independent acquisition (DIA)

LC–MS/MS analyses were performed on a Dionex UltiMate 3000 system online coupled to an Orbitrap Eclipse Tribrid mass spectrometer (Thermo Fisher Scientific, San Jose, CA). The solvent system consisted of 2% ACN, 0.1% FA in water (solvent A) and 98% ACN, 0.1% FA in water (solvent B). Briefly, proteolytic peptides were loaded onto an Acclaim PepMap 100 C18 trap column with a size of 0.1 × 20 mm and 5 µm particle size (Thermo Fisher Scientific) for 5 min at 5 µL/min with 100% solvent A, loading an amount of 800 ng for each of the cohort samples and the unfractionated pool samples, and 400 ng for the HPRP fractions. Peptides were eluted on an Acclaim PepMap 100 C18 analytical column sized as follows: 75 µm × 50 cm, 3 µm particle size (Thermo Fisher Scientific) at 0.3 µL/min using the following gradient of solvent B: 2% for 5 min, linear from 2 to 25% over 96 min, linear from 25 to 40% over 23 min, linear from 40 to 50% over 6 min, and up to 80% in 1 min with a total gradient length of 170 min.

All samples—for the generation of the spectral library and for the final quantitative analysis of the cohort samples—were analyzed in DIA mode. Full MS spectra were collected at 120,000 resolution (AGC target: 3e6 ions, maximum injection time: 60 ms, 350–1650 m/z), and MS2 spectra at 30,000 resolution (AGC target: 3e6 ions, maximum injection time: Auto, NCE: 27, fixed first mass 200 m/z). The DIA precursor ion isolation scheme consisted of 26 variable windows covering the 350–1650 m/z mass range with an overlap of 1 m/z (Additional file 12: Table S11) [41].

### Data analysis

#### Spectral library generation

The DIA spectral library was generated directly in Spectronaut (version 15.1.210713.50606; Biognosys) using Biognosys (BGS) default settings and a mouse UniProtKB-TrEMBL database (86,521 entries, release 08/2021). Briefly, for the Pulsar search, trypsin/P was set as the digestion enzyme and 2 missed cleavages were allowed. Cysteine carbamidomethylation was set as fixed modification, and methionine oxidation and protein N-terminus acetylation as variable modifications. Identifications were validated using 1% false discovery rate (FDR) at the peptide spectrum match (PSM), peptide and protein levels, and the best 3–6 fragments per peptide were kept. The final spectral library contains 46,185 modified peptides and 5,363 protein groups, and can be found in Additional file 7: Table S6.

#### DIA data processing and statistical analysis

DIA data were processed in Spectronaut (version 15.1.210713.50606) using the previously described library. Data extraction parameters were set as dynamic and non-linear iRT calibration with precision iRT was selected. Identification was performed using a precursor PEP cut-off of 0.2 and a 1% precursor and protein q-value (experiment). Quantification was based on MS2 area, and local normalization was applied. Differential protein expression analysis was performed using paired t-test, and p-values were corrected for multiple testing, specifically applying group wise testing corrections using the Storey method [42]. Protein groups with at least two unique peptides, q-value < 0.05, and absolute log_2_(fold-change) > 0.2 were considered to be differentially expressed (Additional file 7: Table S6).

### Statistical processing

Partial least squares-discriminant analysis (PLS-DA) of the proteomics data was performed using the package mixOmics [43] in R (version 4.0.2; RStudio, version 1.3.1093).

### Pathway analysis

An over-representation analysis (ORA) was performed using Consensus Path DB-mouse (Release MM11, 14.10.2021) [44, 45] to evaluate which gene ontology terms were significantly enriched. Gene ontology terms identified from the ORA were subjected to the following filters: q-value < 0.05, term category = b (biological process), and term level > 1.

### Microglia RNA-seq

#### Tissue dissociation

Striatal cell isolation was performed as previously described [46]. Briefly, animals were deeply anesthetized with an intraperitoneal injection of pentobarbital (50 mg/kg IP) and transcardially perfused with 15 ml ice-cold, calcium- and magnesium-free phosphate-buffered saline PBS, pH 7.3–7.4. The brains were quickly removed and striata were dissected on a cooled petri dish and placed in ice-cold Hibernate-A medium. Striata from each mouse were gently dissociated mechanically in Hibernate-A medium in a 1 mL Dounce homogenizer using the loose pestle. The homogenized tissue was then sieved through a 70 μm cell strainer and transferred to a 15 mL falcon tube. The homogenates were pelleted at 450x*g* for 6 min at 4 °C. Pellets were resuspended in PBS to a final volume of 1.5 mL. 500 μL of freshly prepared isotonic percoll solution (pH 7.4) was then added to each sample (final volume: 2 mL) and mixed well. Percoll was rendered isotonic by mixing 1 part of 10 × PBS (pH 7.3–7.4) with 9-parts of percoll. The percoll solution was mixed properly with the cell suspension, after which 2 mL of PBS were gently layered on top of it creating two separate layers. The samples were centrifuged for 10 min at 3000x*g*. The upper layers were aspirated, leaving about 500 μL as some cells float in percoll just above the pellet. The cells were then washed once in PBS making sure not to resuspend the pellet. This was achieved by gently adding 4 mL PBS, closing the tube, and holding it in a horizontal position, and gently tilting it 145 degrees in order to mix the remaining percoll with the added PBS. The cells were then pelleted by centrifuging them at 450x*g* for 10 min at 4 °C. The resulting total striatal cell pellet was used for the magnetic sorting of microglia as described below.

#### Microglia isolation

Microglia were isolated from 1 striatum/sample via magnetic-activated cell sorting (MACS) using mouse anti-CD11b (for microglia) paramagnetic nanobeads (Miltenyi) according to the manufacturer’s instructions with some modifications. The MACS buffer used consisted of 1.5% bovine serum albumin (BSA) diluted in PBS from a commercial 7.5% cell-culture grade BSA stock (Thermo Fisher Scientific). For the microglial isolation, total striatal cell pellets after percoll (see above) were re-suspended in 90 μL MACS buffer and 10 CD11b beads (Miltenyi). Cells were then incubated for 15 min at 4 °C. Excess beads were washed with 1 mL MACS buffer and the cells pelleted at 300 rcf (relative centrifugal force) for 5 min at 4 °C. The cells were then passed through an MS MACS column attached to a magnet whereby CD11b positive cells stay attached to the column, whereas unlabeled cells flowed through the column. After washing the columns three times with MACS buffer, microglia were flushed from the column with 1 mL MACS buffer and pelleted at 300 rcf for 5 min at 4 °C. Cell pellets were lysed in QIAzol (Qiagen product code 79306), snap-frozen in dry ice and stored at -80 °C until RNA extraction.

### Western blot

Flash frozen striatal samples were homogenized in a RIPA buffer (Pierce 89900) containing freshly added phosphatase (Pierce) and protease (Roche) inhibitors, centrifuged for 20 min at 15,000 x*g* and the supernatant was collected. Protein concentration was determined using the BCA method. For each sample, 20 μg of protein was resolved in 4–12% Bis/Tris-acrylamide gradient gels (BioRad) and transferred to nitrocellulose membranes. Membranes were incubated with the following primary antibodies: anti-PSD-95 clone 6G6-1C9 (1:1000, MAB1596, Millipore), anti-synaptophysin (1:500, ab16659, Abcam), anti-phospho-p44/42 MAPK (Erk1/2) (Thr202/Tyr204) (1:1000, #9101, Cell Signaling), anti-p44/42 MAPK (Erk1/2) Antibody (1:1000, #9102, Cell Signaling), anti-C1q [4.8] (1:500, ab182451, Abacam), anti-DARPP-32 (1:1000, #2306, Cell Signaling), anti-Iba1 (1:1000, 016-20001, Wako), anti-Htt (1:1,000; Millipore EM48 MAB5374) and anti-GAPDH (D16H11) (1:2000, #5174, Cell Signaling) antibodies. The secondary HRP conjugated antibodies included anti-rabbit (1:2000, PI-1000, Vector laboratories) and anti-mouse (1:2000, PI-2000, Vector laboratories). Following development with ECL (Pierce®) pictures were acquired using a Fujifilm ImageReader LAS-4000, and bands were quantified using ImageJ (Fiji software package). Protein levels were normalized to GAPDH, and mean values (Ns specified in the figure legend) were normalized to Control = 100.

### Behavior

*Accelerating rotarod*. Motor learning was evaluated as in [47, 48]. With neither training nor habituation sessions, mice were placed on a motorized rod (3 cm diameter) with a gradual speed increase from 4 to 40 RPMs for 5 min. Each mouse was evaluated over three days, conducting three trials per day, with an intertrial interval of 1 h. Latency to fall was recorded. Trials were included only if the mouse placed all four paws on the rod.

*Balance beam test*. Balance was evaluated as in [47, 48]. The beam consisted of an 85 cm long wooden prism, divided into 5 cm frames, with a 1 cm face, placed 40 cm above the bench surface. The test consisted of two sessions, the training and the testing, separated by 4 h. In both training and testing sessions, mice walked along the beam for 2 min. During the testing session latency to cover 30 frames and total distance traveled were measured.

*Vertical pole test*. Balance was evaluated as in [47, 48]. The pole test consisted of a 60 cm wooden cylinder (1 cm diameter) wrapped in tape to facilitate walking. Mice were trained for two consecutive days and tested on the third day. Three trials per session were conducted. Mice were placed with heads facing upward just below the top of the pole. Both time to complete a turn, in other words, orient the body downward, and time to climb down (time to descend) the pole were measured. Trials in which mice descended the pole without complete reorientation were counted as error trials and analyzed separately.

### Statistical analysis

The non-genomic data (Figs. 2, 3, 4, 6G, 7, 8J) were analyzed with GraphPad Prism 8. Graphs represent the mean of all samples in each group ± SEM. Sample sizes (n values) and statistical tests are indicated in the figure legends. A two-way ANOVA followed by a Bonferroni’s post-hoc test was used for multiple comparisons. A Student’s t-test was used for simple comparisons. Significance is reported at *p < 0.05, **p < 0.01, ***p < 0.001.

## Results

### *TYROBP* network is overactivated in human HD putamen

**Fig. 1:**
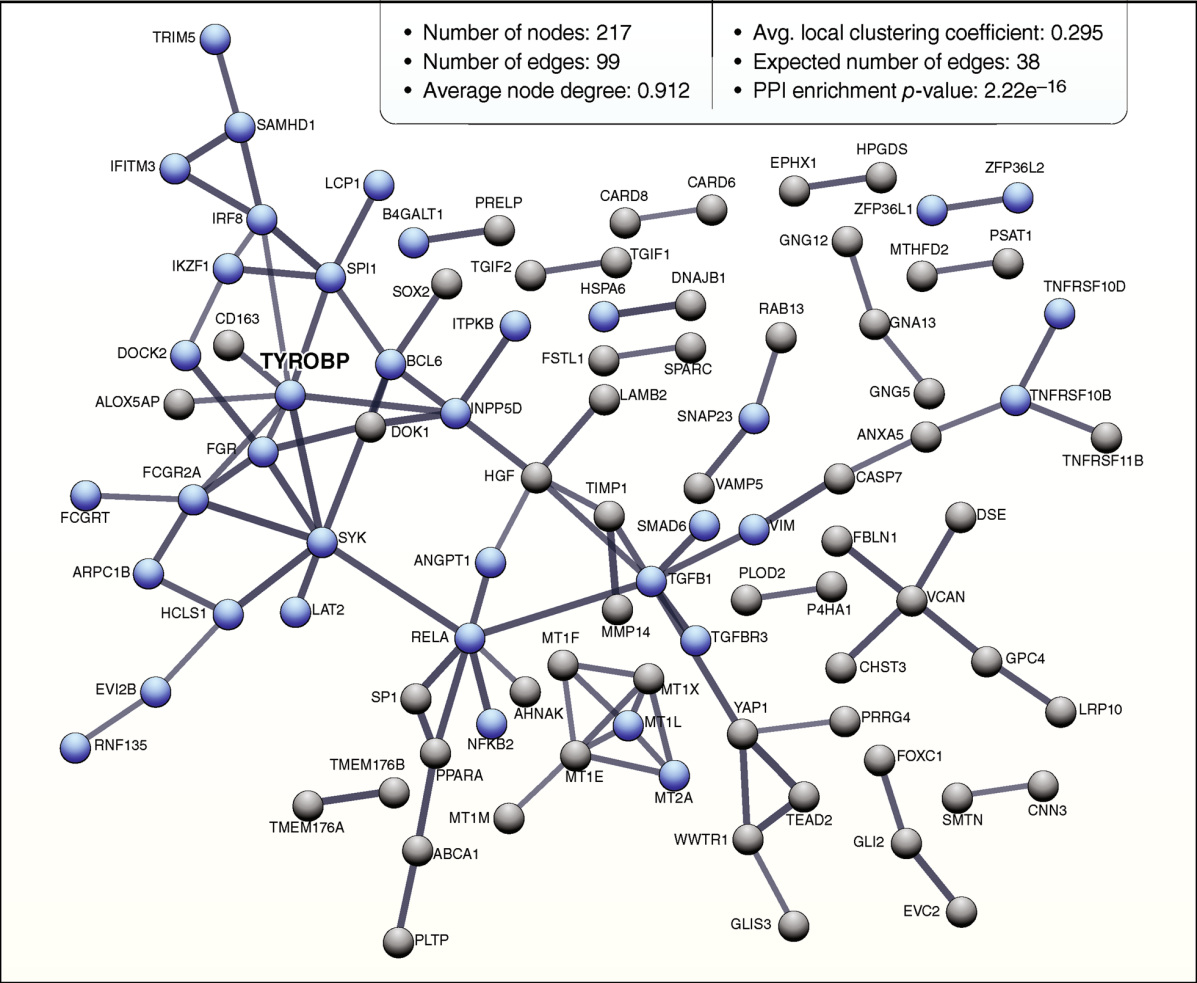
*TYROBP* network is upregulated in HD brains. STRING-generated interaction network of the common differentially expressed genes (DEGs) detected by RNA-seq in HD brains. STRING v11.5 was used to derive the network of 221 DEGs applying following prediction methods: text mining, experiments, databases, co-expression, neighborhood, gene fusion, co-occurrence. The nodes that did not interact with other nodes were deleted. The blue nodes illustrate genes/proteins assigned to “immune system process” Gene Ontology pathway. Line thickness indicates the strength of data support. Interaction score > 0.7

In HD transcriptomics, down-regulated genes and pathways are predominantly neuronal, and in the striatum, expressed in medium spiny neurons (MSNs). To determine which pathways are up-regulated, or activated in HD brains, we performed a comprehensive review of publicly available human gene expression datasets, with a focus on upregulated differentially expressed genes (DEGs). We observed a distinct pattern of gene expression that was consistent across multiple datasets [24, 28], with upregulated genes dominated by those involved in immune and inflammatory-related processes, importantly including the *TYROBP* network in microglia. Microarray datasets revealed DEGs associated with Hedgehog signaling pathway, EGF/EGFR Signaling Pathway and focal adhesion, including integrin-encoding genes, which are possibly related to microglial motility and activation (Table 1). Enrichment analysis results obtained from each dataset are shown in Additional file 2: Table S1. We next identified the genes that are commonly upregulated across all HD RNAseq datasets, resulting in a total of 221 genes. To inquire into the role of commonly upregulated DEGs across HD brains, we analyzed them by means of the STRING v11.5 tool. The analysis generated a gene/protein interaction network (Fig. 1; strength of interaction score > 0.7) consisting of 217 DEGs (nodes) and 99 edges. Nodes devoid of any interactions were deleted from the network. With 8 edges, *TYROBP* is the most interactive node, followed by *RELA*, *SYK* and *TGFB1* with 7 edges each. GO terms associated with the network are related to response to stimulus and immune system process (Additional file 3: Table S2). Functionally related significant modules from the network were mined with MCODE with cutoffs of the MCODE score ≥ 2 and number of nodes ≥ 2. Three significant modules were detected, and the top module with MCORE score 4.5 (5 nodes, 9 edges) was formed by metallothionein-related proteins. The second most significant module with MCORE score 4.0 (4 nodes, 6 edges) contained *TYROBP*, *SYK*, *FGR* and *FCGR2A*. Importantly, TYROBP signals through SYK to activate intracellular pathways including extracellular signal-regulated protein kinase (Erk), phosphatidylinositol 3-kinase (PI3K), phospholipase Cγ (PLCγ), and Vav [49]. SYK is rapidly becoming a target of interest for AD therapeutics [50–53]. Together, these data suggest that *TYROBP* may play an important role in the pathophysiologic process in HD.

**Table 1:** TYROBP network is upregulated in HD brains

GSE Citation	Assay	Brain region	#DEGS	Enriched wikipathways	# Genes TYROBP Network
**PRJEB44140** Elorza et al., 2021	RNA-seq	**Striatum**	2257	IL-18 Signaling Pathway; VEGFA-VEGFR2 Signaling Pathway; Apoptosis; **TYROBP Causal Network in Microglia**; Apoptosis Modulation and Signaling	**25**
**GSE64810** Labadorf et al., 2015	RNA-seq	**BA9**	1430	**TYROBP Causal Network in Microglia**; Complement and Coagulation Cascades; Complement System Microglia Pathogen Phagocytosis Pathway; Prostaglandin Synthesis and Regulation	**30**
Al-Dalahmah et al., 2020	RNA-seq	**Cingulate cortex**	1085	**TYROBP Causal Network in Microglia**; Vitamin D Receptor Pathway; Ebola Virus Pathway on Host; Hippo-Merlin Signaling Dysregulation; Platelet-mediated Interactions with Vascular and Circulating Cells	**25**
**GSE26927** Durenberger et al., 2015	Microarray	**Caudate**	659	Hedgehog Signaling Pathway Netpath; Hedgehog Signaling Pathway; Non-genomic Actions of 1,25 dihydroxyvitamin D3; Hippo-Merlin Signaling Dysregulation; Primary Focal Segmental Glomerulosclerosis	**2**
**GSE3790** Hodges et al., 2006	Microarray	**Caudate**	3289*	EGF/EGFR Signaling Pathway; Acute Viral Myocarditis; Small Lung Cell Cancer; Focal Adhesion; Apoptosis	**19**
		**BA4**	326*	Zinc Homeostasis; Copper Homeostasis; Nanomaterial-induced Apoptosis	**0**
		**Cerebellum**	101*	Hippo-Merlin Signaling Dysregulation; ERK Pathway in Huntington’s Disease; PI3K-Akt Signaling Pathway; Brain-derived Neurotrophic Factor (BDNF) Signaling Pathway; Focal Adhesion-PI3K-Akt-mTOR-signaling Pathway	**0**

Overview of the published datasets used in this study. *nominal p-value < 0.001 was used as threshold. Upregulated genes dominated by those involved in immune and inflammatory-related processes, importantly including the *TYROBP* network in microglia

### *Tyrobp* deletion prevents pro-inflammatory phenotype of Q175 microglia

**Fig. 2:**
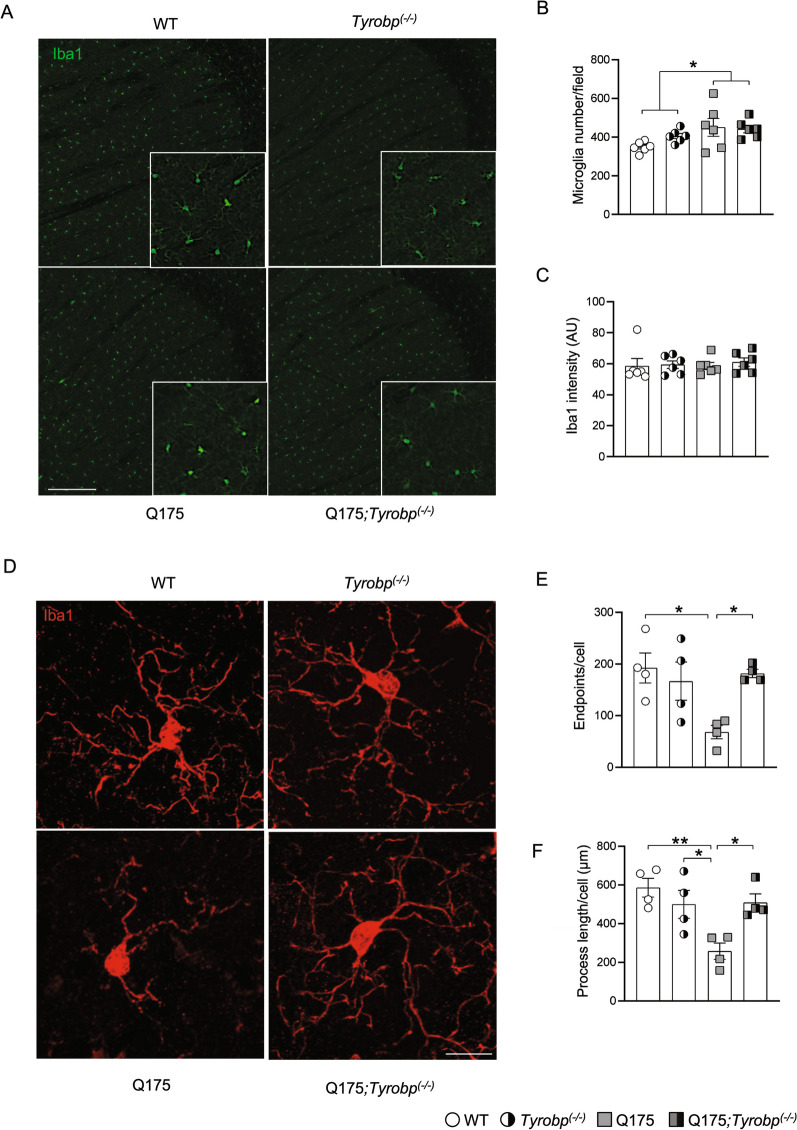
*Tyrobp* deletion corrects microglia morphologic parameters. **A** Representative Iba1 immunofluorescence (20X) in the striatum of a 10-month old WT or Q175 mouse with (left) and without (right) *Tyrobp*. Scale bar: 300 µm. Quantification of Iba1-immunopositive **B** cell number and **C** intensity in striatal area (n = 6 mice per group with an average of 3 slices per mouse). **D** High magnification illustration of Iba-1 immunostaining images of the striatum from 10-month old WT and Q175 mice with (left) and without (right) *Tyrobp*. Scale bar: 15 µm. **E** Endpoints/cell and **F** process length of striatal microglia. n = 15 microglia per mouse with n = 4 mice per group. Error bars represent means ± SEM. Males and females were used for experiments, and results were combined for analysis. Statistical analyses were performed using a Two-Way ANOVA with Bonferroni as post-hoc test. *p < 0.05; **p < 0.01

We previously reported that deletion of *Tyrobp* altered microglial response to AD-related pathologies, including amyloidopathy and tauopathy [28–30]. Microgliosis and neuroinflammation are evident in HD human caudate/putamen beginning at presymptomatic stages [11]. Our in-silico analysis pointed to a key role of *TYROBP* in HD pathophysiology, so we performed a mouse genetics analysis, taking advantage of a previously generated *Tyrobp*-null [*Tyrobp* homozygous knockout (*Tyrobp*^*(−/−)*^)] mouse line [36]. *Tyrobp-*null mice were bred with Q175 heterozygous mice to analyze Q175 mice on a *Tyrobp*^*(−/−)*^ background (See schematic, Additional file 1: Fig. S1A). The Q175 model is a genetically accurate mouse model of adult-onset HD, in which human *HTT* exon 1 (with a ~ 190 CAG repeats) was knocked into the endogenous *Htt*. This well-defined model recapitulates many of the molecular, neuropathological, and behavioral abnormalities observed in HD [54]. We evaluated the effects of *Tyrobp* deletion on microglial number in Q175 mice at 10 months of age, a fully symptomatic time-point. Microglia were increased in number in the striatum of Q175 mice, independent of *Tyrobp* expression (Fig. 2A,B). We did not detect changes in Iba1 fluorescence intensity (Fig. 2C), nor mRNA or protein levels (Additional file 1: Fig. S1B). Morphologically, microglial cells from Q175 mice showed decreased branch numbers and total branch length (Fig. 2D–F), indicative of a pro-inflammatory status [55]. Notably, microglia morphology from Q175;*Tyrobp*^*(−/−)*^ mice was similar to the WT microglia (Fig. 2D–F). These data show that *Tyrobp* deletion in Q175 mice prevents HD-associated microglia morphological changes.

### *Tyrobp* deletion reduces microglia CD68 content and impedes PSD-95 loss in Q175 mice

**Fig. 3:**
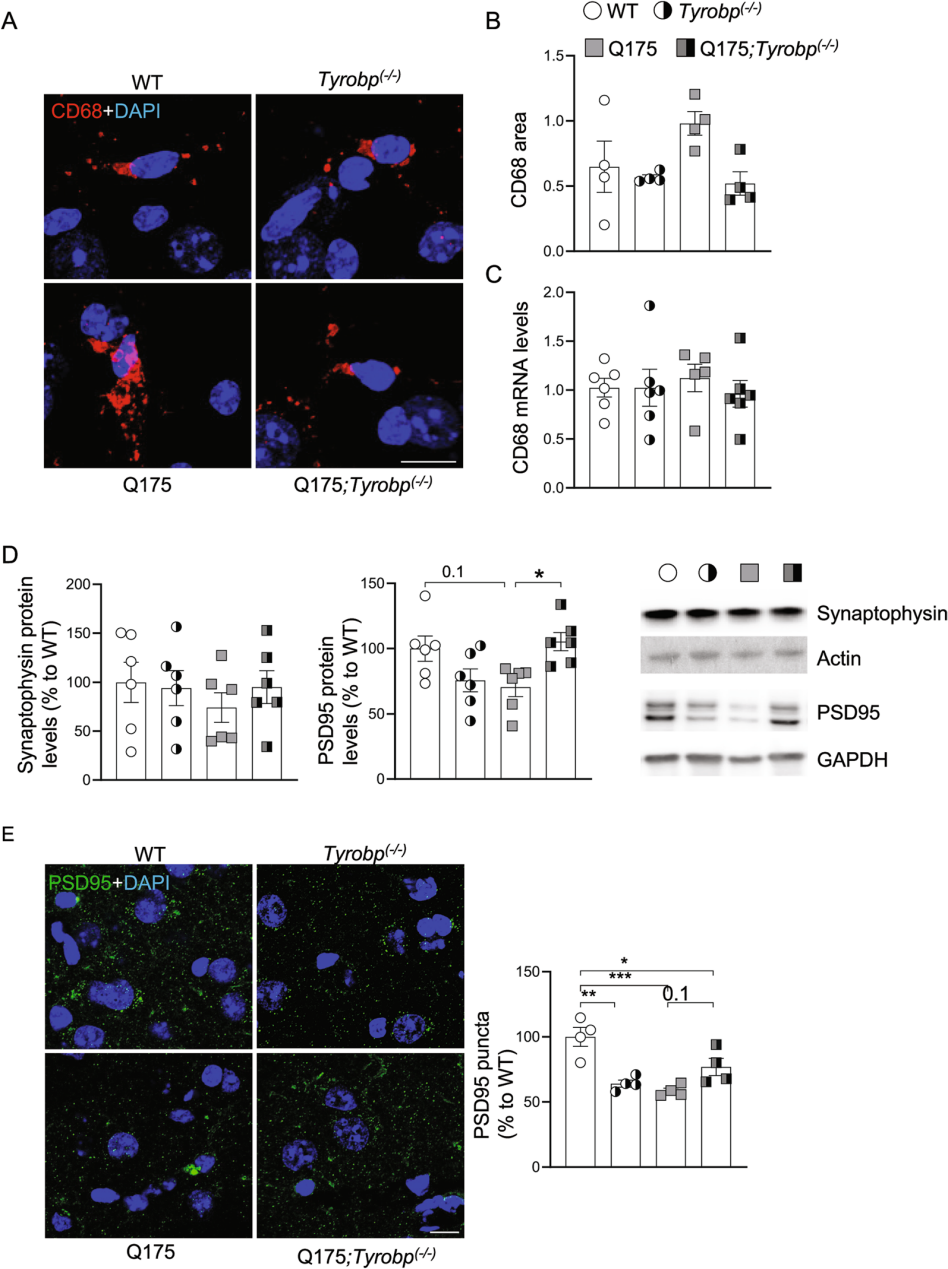
*Tyrobp* deletion prevents increased CD68 levels and reduction of PSD-95 in Q175 mice. **A** Representative images of CD68 immunofluorescent staining in the striatum from 10-month old WT and Q175 mice with (left) and without (right) *Tyrobp*. Scale bar: 10 µm. **B** Quantification of CD68-immunopositive area in the striatum (n = 4 mice per group with an average of 3 slices per mouse). **C** RT-qPCR of CD68 mRNA in the striatum in the same mice as in Fig. 2A with n = 6 mice per group. **D** Western blot and densitometric analysis of synaptophysin and PSD-95 proteins in striatal samples. n = 6 mice per group. (**E)** Representative confocal images showing PSD-95 (green) immunopositive clusters in the dorsal striatum of WT and Q175 mice with and without *Tyrobp*. Quantitative analysis is shown as mean ± SEM (n = 4 animals per group). Statistical analysis was performed using Two-Way ANOVA. *p < 0.05; **p < 0.01; ***p < 0.001. Scale bars, 10 µm

Activated microglia are characterized by increased phagocytic activity [56], and CD68 is a transmembrane protein highly expressed in phagocytic lysosomes of microglial cells [57, 58]. CD68 levels are elevated in the early disease stage of R6/2 mice, a transgenic mouse model of HD with rapid progression [59]. To determine the functional implications of the improved morphological indices in Q175;*Tyrobp*^*(−/−)*^ mice, we evaluated the area covered by CD68 in striatal microglia. *Tyrobp* deletion in Q175 mice normalized striatal CD68 content (Fig. 3A, B; Q175 vs Q175;*Tyrobp*^*(−/−)*^, p = 0.01, 2-tailed t-test). Of note, striatal CD68 mRNA levels were not altered, suggesting that CD68 levels are modulated through post-transcriptional mechanisms (Fig. 3C). Further, the number of CD68-positive cells colabeling with Iba1 were unchanged (Additional file 1: Fig. S2). To determine if deletion of *Tyrobp* impacted levels of synaptic markers, as synaptic deficits may exist in neurodegenerative diseases [60, 61], we assayed synaptophysin and post-synaptic density 95 (PSD-95) protein levels and estimated the density of PSD-95 immunopositive puncta in striatum in the four groups of mice. In agreement with previous reports [58, 62–64], PSD-95 protein levels were reduced in the striatum of Q175 mice (Fig. 3D), which importantly, was prevented in the presence of deletion of *Tyrobp* in Q175 mice (Fig. 3D). There is a trend towards decreased PSD-95 in the *Tyrobp*-null mouse, supporting a role for this gene, and microglia in general, in development and maintenance of synaptic integrity under baseline conditions [65, 66]. Our data would perhaps indicate a specific role for TYROBP in the striatum. In addition, immunostaining revealed that *Tyrobp* deletion in Q175 mice prevented the reduction in PSD-95 puncta (Fig. 3E).

### *Tyrobp* deletion prevents impaired motor function in 6- and 9-month-old Q175 mice

**Fig. 4:**
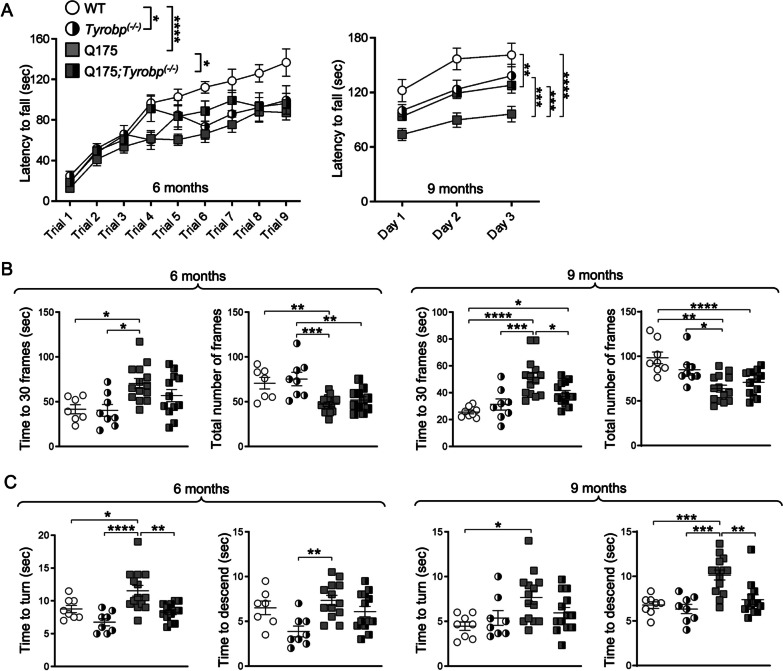
*Tyrobp* deletion ameliorates aspects of the Q175 motor phenotype. Motor behavior in Q175 mice was assessed at 6 and 9 months of age. **A** Accelerating rotarod test was performed for three consecutive days (three trials per day). The latency to fall data at 6 months is represented per test and group as mean ± SEM. The latency to fall at 9 months is averaged per day (WT n = 8; *Tyrobp*^*(−/−)*^ n = 8; Q175 n = 13; Q175;*Tyrobp*^*(−/−)*^ = 12). **B** Balance beam assay is shown as time to cross 30 frames and number of frames crossed in 2 min (WT n = 7; *Tyrobp*^*(−/−)*^ n = 8; Q175 n = 14; Q175;*Tyrobp*^*(−/−)*^ = 13). **C** Vertical pole assay is shown as time to turn (left) and time to descend (right), which were recorded after placing the mice upwards to the pole. Three trials were conducted (WT n = 8; *Tyrobp*^*(−/−)*^; n = 8; Q175 n = 14; Q175;*Tyrobp*^*(−/−)*^ = 13). Data represent the mean ± SEM. Each point represents data from an individual mouse. Statistical analysis was performed using Two-way ANOVA followed by Bonferroni’s post hoc test, *p < 0.05; **p < 0.01; ***p < 0.001, ****p < 0.0001

Next, we investigated whether absence of *Tyrobp* modulates motor phenotype in the Q175 mouse model. Motor coordination was assessed by the accelerating rotarod, which also evaluates motor learning, and is highly sensitive to subtle motor changes. Balance was evaluated using the balance beam and vertical pole tests. To determine if *Tyrobp* deletion modifies the disease onset and/or improves motor behavior at symptomatic stages, motor performance was examined at two time points, 6 and 9 months of age. In the majority of the motor assays, *Tyrobp* deficiency alone does not have a significant effect on the behavior assay. The exception is that, at 6 months, *Tyrobp*-null mice do indeed demonstrate a motor learning deficit in the rotarod assay, again suggesting a role for *Tyrobp* in development and maintenance of striatal function. *Tyrobp* deletion in Q175 mice improved motor learning at both time points (Fig. 4A). Strikingly, balance deficits were detected in Q175, but not Q175;*Tyrobp*^*(−/−)*^ mice at 6 months of age (Fig. 4B, C). We also detected an overall balance improvement in Q175;*Tyrobp*^*(−/−)*^ mice at 9 months of age (Fig. 4B, C). These behavioral data are compatible with a beneficial effect of the *Tyrobp* deletion on the Q175/HD motor phenotype. Notably, a decrease restricted to C1Q level did not report an improvement in motor performance in the same HD model [61].

Importantly, deletion of *Tyrobp* had no effect on the number of mHtt inclusions in the dorsal striatum (Additional file 1: Fig. S3). In the Novel Object Recognition assay, both WT and *Tyrobp*-null mice spent more time exploring the novel object, whereas Q175 mice spent equal time exploring the old and novel object, without improvement in the absence of *Tyrobp* (Additional file 1: Fig. S4). Interestingly, this is in contrast to what was observed in R6/2 mice following microglial ablation [21], suggesting that *Tyrobp* deletion does not correct all the effects of microglia in an HD model. There is of course a major caveat in that Q175 and R6/2 are very different models.

### *Tyrobp* deletion in HD mice normalizes human-specific pro-inflammatory pathways

**Fig. 5:**
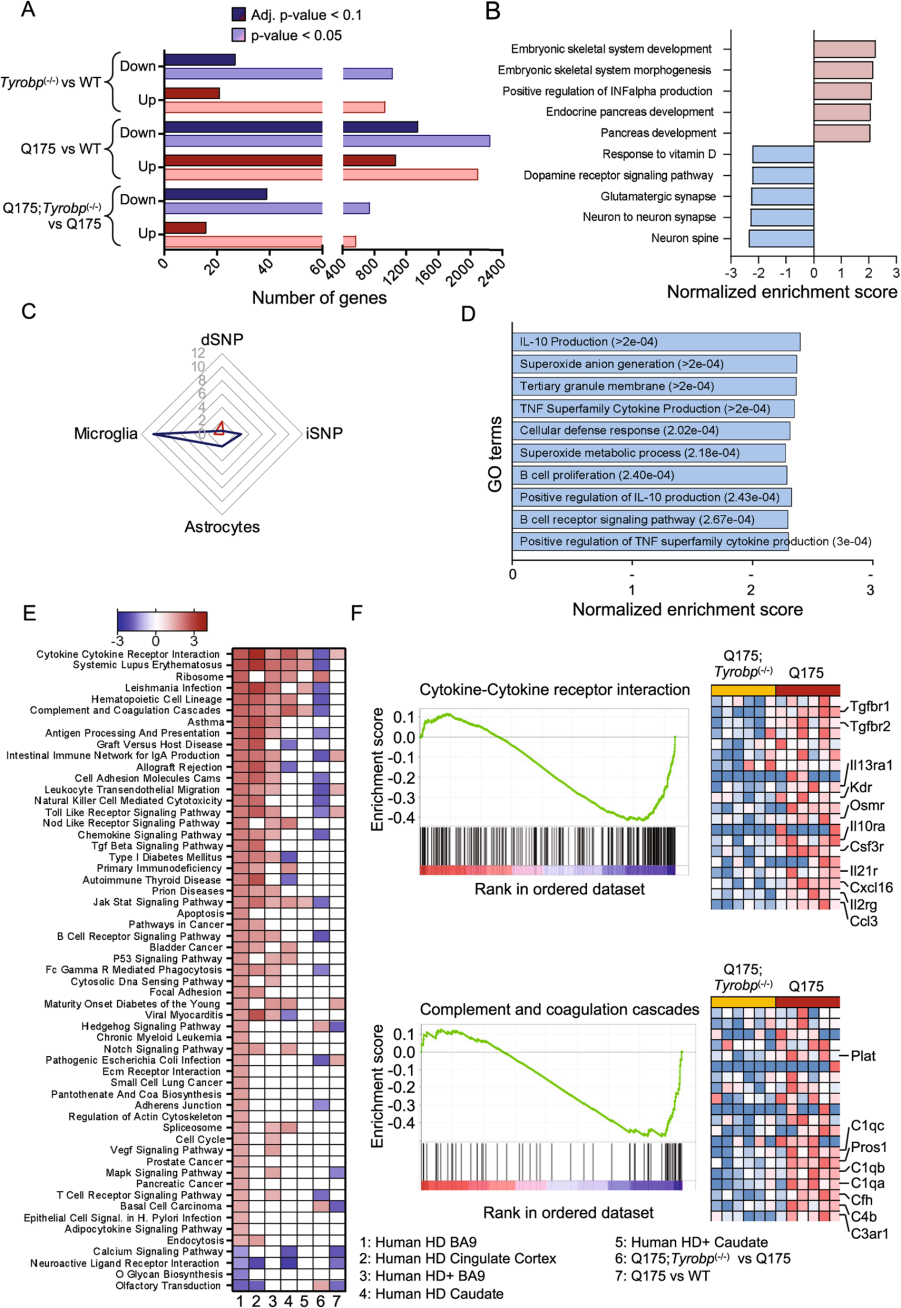
*Tyrobp* deletion reduces pathways activated in human HD brain but not in HD mouse models. Bulk RNA-seq was performed on whole striatal RNA. **A** The number of DEGs for each data set at adjusted p < 0.1 and nominal p < 0.05 is shown. Blue bars indicate downregulated DEGs, and red bars indicate upregulated DEGs. **B** GO terms (biological process) associated with genes from Q175 vs WT data sets after GSEA analysis. **C** Cell-type enrichment analysis for Q175; *Tyrobp*^(−/−)^ vs Q175 DEGs (nominal p-value < 0.05). Lines represent − log_10_ p-value of chi-square calculation of the cell type enrichment. **D** GO terms (biological process) associated to genes from Q175;*Tyrobp*^(−/−)^ vs Q175 data sets after GSEA analysis. dSNP = direct (Drd1) spiny neurons; iSNP = indirect (Drd2) spiny neurons (**E**) Heatmap of Normalized Enrichment Score for Kyoto Encyclopedia of Genes and Genomes (KEGG) pathways detected in human and mouse datasets. Pathways have been ranked based on descending normalized enrichment score from the Human HD BA9 dataset (Agus et al., 2019 [15]). Column #1: Human HD Brodmann area 9 (BA9) (Agus et al., 2019 [15]); #2: Human HD Cingulate Cortex (Al-dalahmah et al., 2020 [33]); #3 Presymptomatic human HD BA9 vs control (Agus et al., 2019 [15]); #4: Human HD caudate (CAU) v Control (Elorza et al., 2021 [31]); #5 Presymptomatic Human Caudate (Agus et al., 2019 [15]); #6: Q175;*Tyrobp*^(−/−)^ vs Q175; #7: Q175 vs WT. **F** GSEA results for Cytokine-Cytokine receptor interactions and Complement and coagulation cascade pathways. Normalized gene scores are shown

Deletion of *Tyrobp* in AD mouse models corrects many microglia-related transcriptomic abnormalities, mainly by repressing the expression of disease-associated microglia (DAM)-related genes [28]. We therefore sought to determine whether *Tyrobp* deletion would restore the overall transcriptome of HD models and/or correct pathways altered in HD human brain. We performed bulk RNA-seq analysis on striatal samples from the 10-month-old WT and Q175 mice which were tested with motor assays, including WT or homozygous KO for *Tyrobp*. Consistent with results from previous studies [18, 67–73], Q175 mice showed marked transcriptomic abnormalities, with a total of 2413 differentially expressed genes (DEGs) (false discovery rate (FDR) < 0.1) relative to WT mice. Of these, 1069 were upregulated and 1344 downregulated (Fig. 5A). As expected and previously reported, we detected strong downregulation of MSN identity genes, including *Drd1*, *Drd2*, *Ppp1r1b* and *Adora2a* (Additional file 4: Table S3). We performed gene ontology analysis using gene set enrichment analysis (GSEA). The HD transcriptome showed strong downregulation of neuronal-related genes and overall activation of development-related genes (Fig. 5B; Additional file 4: Table S3; Additional file 5: Table S4).

*Tyrobp* deletion in Q175 mice induced small transcriptomic changes relative to Q175 alone, with 55 DEGs, of which 16 were upregulated and 39 downregulated. At a lower stringency threshold (p-value < 0.05), we detected more than 1000 DEGs (Fig. 5A; Additional file 4: Table S3). We confirmed the markedly decreased expression of *Tyrobp*, and importantly, downregulated DEGs are associated with microglial cells (Fig. 5C). We again used GSEA to identify which biological processes and pathways were affected by *Tyrobp* deletion in Q175 mice. The most significantly downregulated biological processes were IL-10 production, superoxide anion generation, and TNF superfamily cytokine production which includes, among other genes, *Clec7a*, *Trem2* and *Tlr4* (Fig. 5D; Additional file 5: Table S4). Those biological terms are related to immune and inflammatory responses. Both *TREM2*, an important component of the DAM phenotype which signals via TYROBP and is an AD GWAS risk factor gene, and *TLR4* are increased in the putamen of HD patients and, importantly, are genetic modifiers of HD. The *TREM2* R47H gene variant is associated with changes in cognitive decline in HD patients, and rs1927911 and rs10116253 *TLR4* single nucleotide polymorphisms are associated with the rate of motor decline [74]. As previously stated, genes associated with neuroinflammatory and neuroimmune responses are upregulated in the striatum and cortex of human HD brains beginning at presymptomatic stages [15], but these neuroinflammatory genes are unchanged from WT in the analysis of Q175 mice presented herein.

To evaluate if *Tyrobp* deletion modulates pathways selectively activated in HD human brains which may not be dysregulated in the mouse, we took advantage of previously published pathway analysis performed on transcriptomic data obtained from HD human presymptomatic caudate, and symptomatic and presymptomatic cortex [15]. We also performed gene set enrichment analysis on the RNA-seq datasets used in Fig. 1 (Additional file 6: Table S5). The key finding is that the human pro-inflammatory gene signature is downregulated in Q175/*Tyrobp*^*(−/−)*^ mice (Fig. 5D) even though not originally increased in Q175. Pathways activated in symptomatic and presymptomatic human brains that are downregulated by *Tyrobp* deletion in Q175 brains include cytokine-cytokine receptor interaction and complement and coagulation cascades (Fig. 5E, F). Importantly, *Tyrobp* deletion in Q175 mice does not normalize DARPP-32 levels (Additional file 1: Fig. S5) nor the expression of other MSN-specific genes (Additional file 1: Fig. S6), indicating a lack of non-cell-autonomous effects on the MSN neuronal-specific transcriptome. These data imply that *Tyrobp* deletion does not restore neuronal pathways at the gene expression level but may be a novel target by which to reduce the expression of pro-inflammatory pathway-related genes, which alone may mitigate aspects of the mouse HD phenotype.

### *Tyrobp* is a mediator of C1Q level and astrogliosis in Q175 mice as shown by integration of proteomics and transcriptomics data

Thus far, we have identified the overall transcriptomic changes induced by *Tyrobp* deletion in Q175 mice, with a focus on cell-autonomous effects and highlighting the complement system. Next, we used proteomics to validate candidates underlying the amelioration of the Q175 mouse motor phenotype in Q175;*Tyrobp*^*(−/−)*^ mice. We performed a comprehensive and quantitative workflow using data-independent acquisition (DIA) [75–77] on striatal tissue samples from 10-month-old WT, Q175, Q175;*Tyrobp*^*(−/−)*^ and *Tyrobp*^*(−/−)*^ mice. Briefly, for quantification, all peptide samples were analyzed by DIA using variable-width isolation windows. The variable window width is adjusted according to the complexity of the typical MS1 ion current observed within certain m/z. DIA acquisitions produce complex MS/MS spectra, which are a composite of all the analytes within each selected Q1 m/z window, and subsequently, allow for highly comprehensive and accurate quantification and deep coverage. In addition, DIA workflows are not limited by the stochastic peptide MS/MS sampling biases characteristic of traditional data-dependent acquisition (DDA) mass spectrometry [76]. In order to obtain a deep spectral library for our quantitative analysis of mouse striatum from 4 different mouse strains, we first pooled aliquots of proteolytic peptides obtained after Lys-C and trypsin digestions from all samples, and further offline fractionated a subset of the pooled samples using High-pH Reversed-Phase Peptide Fractionation (HPRP). After label-free DIA acquisitions [75–77] of each of the 8 HPRP fractions and several unfractionated pooled samples using the Orbitrap Eclipse Tribrid mass spectrometer, we generated a resource of a striatum-specific spectral library containing 46,185 peptides corresponding to 5363 unique protein groups (7950 proteins).

**Fig. 6:**
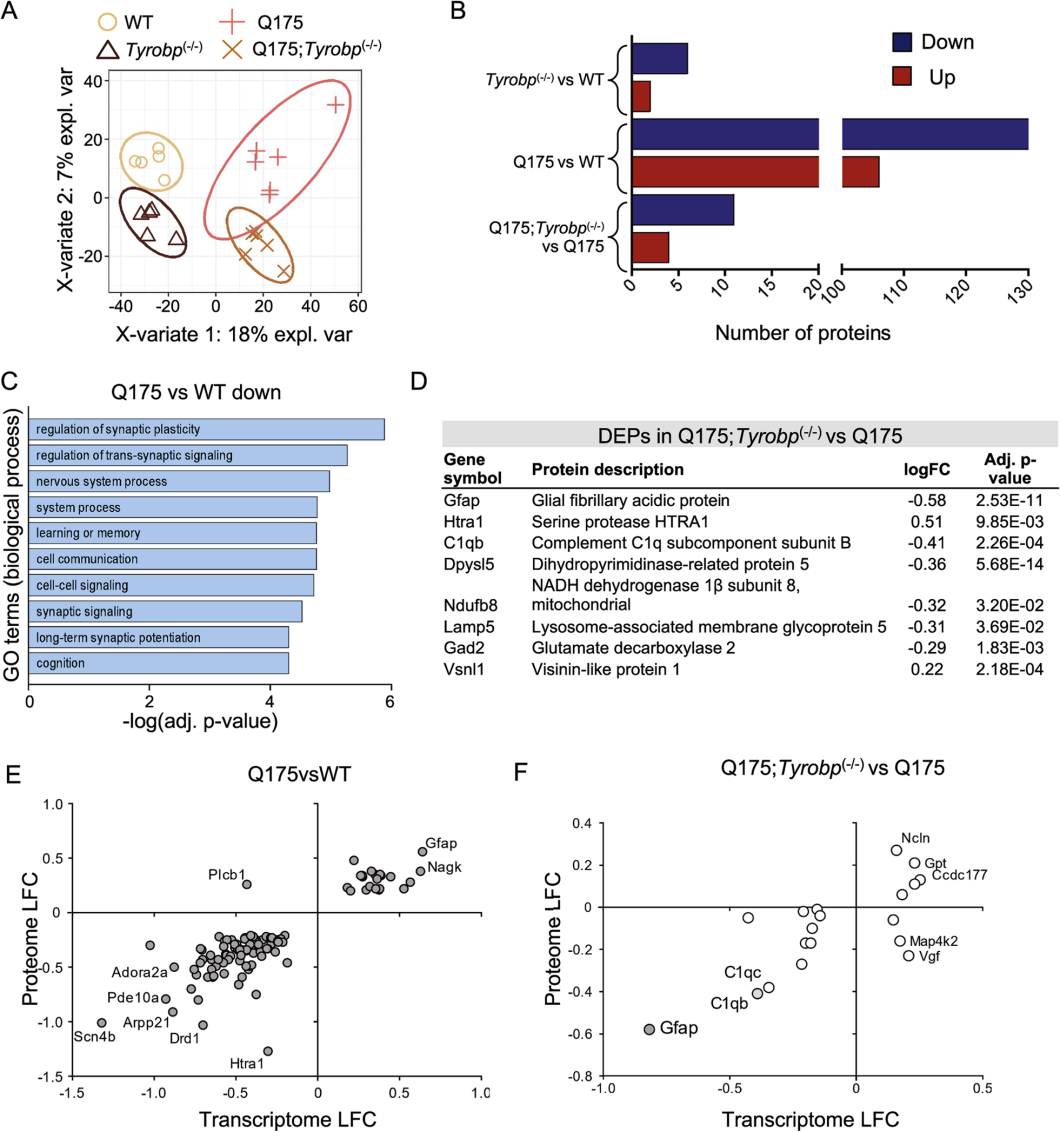
Proteomic analysis reveals reduction of astrogliosis in Q175 mice lacking *Tyrobp*. **A** Supervised Clustering using Partial Least Squares-Discriminant Analysis (PLS-DA). **B** Summary of the number of differentially expressed proteins (FDR < 0.05 and absolute log_2_(fold change) > 0.2). Blue and red bars indicate downregulated and upregulated DEGs, respectively. **C** GO terms (biological process) associated with downregulated proteins from Q175 vs WT data sets after ConsensusPathDB overrepresentation analysis. **D** Top differentially expressed proteins in the striatum of Q175;*Tyrobp*^(−/−)^ vs. Q175 mice. **E**, **F** Plot showing Q175 vs WT (left) and Q175;*Tyrobp*^(−/−)^ vs Q175 (right) log_2_ fold-change (LFC) in the transcriptome and proteome. The plot only includes those genes detected as both transcripts and proteins and also differentially expressed (adj. p-value < 0.1 for Q175 vs WT comparison; and nominal p-value < 0.05 for Q175;*Tyrobp*^(−/−)^ vs Q175 comparison). Gray color denotes that the gene was differentially expressed in the transcriptome and proteome with an adj. p-value < 0.1

**Fig. 7:**
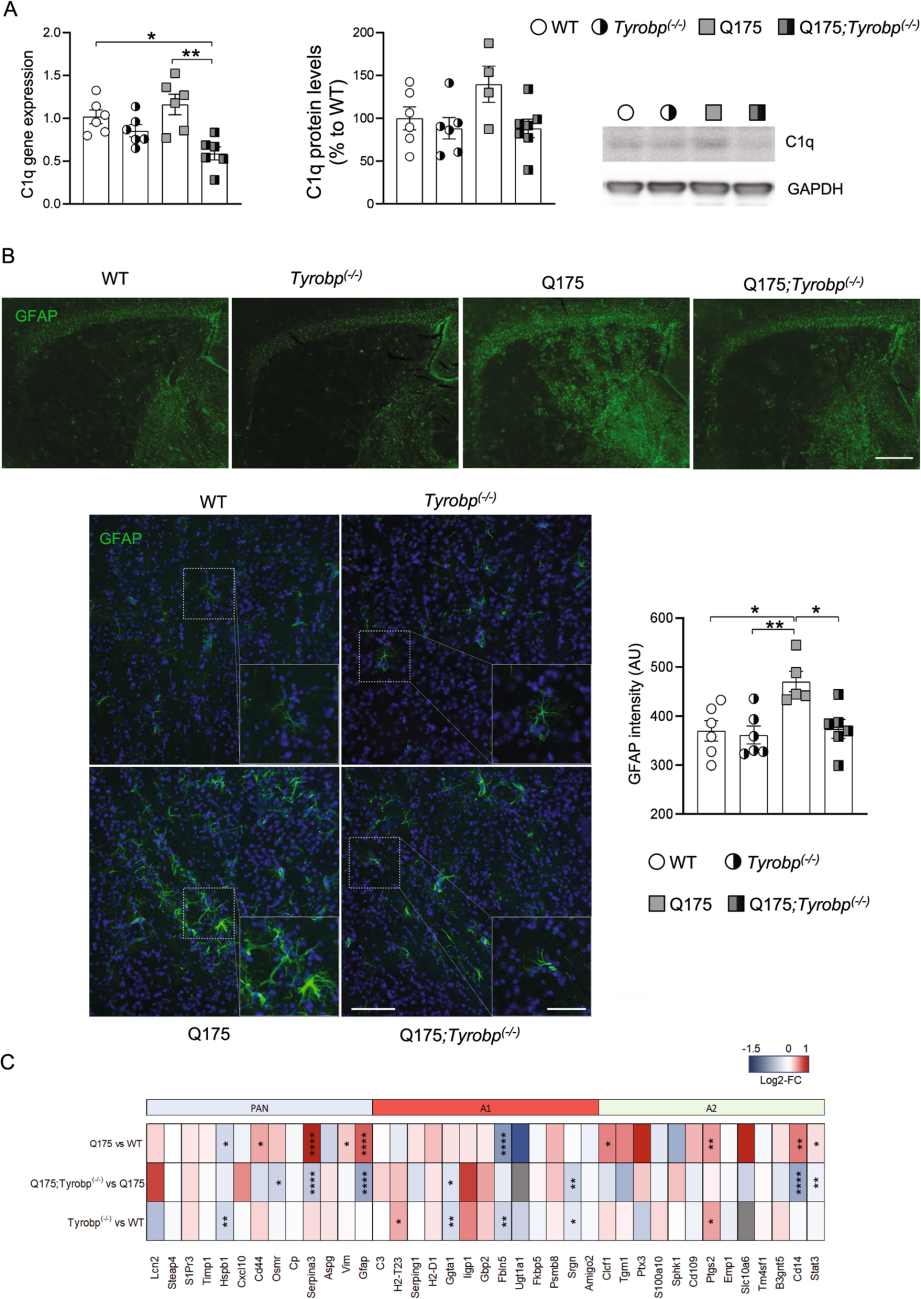
*Tyrobp* deletion reduces C1Q and GFAP levels in Q175 mice. **A** RT-qPCR of *C1q* mRNA and western blot and densitometric analysis of C1Q protein in the striatum of WT and Q175 mice with and without *Tyrobp* (10 months of age), n = 4–6 mice per group. Quantitative analysis is shown as mean ± SEM. Each point represents data from an individual mouse. Statistical analysis was performed using Two-Way ANOVA. *p < 0.05; **p < 0.01. **B** Representative images showing GFAP (green) staining in the striatum of WT and Q175 mice with and without *Tyrobp* (upper panel). 4X magnification, Scale bar, 500 µm. Representative images (20X and inset, 40X) showing GFAP (green) staining in the striatum of WT and Q175 mice with and without *Tyrobp*. Quantification of the intensity is shown as mean ± SEM (n = 5–6 mice per group). Each point represents data from an individual mouse. Statistical analysis was performed using Two-Way ANOVA. *p < 0.05; **p < 0.01. Scale bar, 25 µm. **C** Heatmap showing Q175 vs WT (top) and Q175;Tyrobp(−/−) vs Q175 (middle) and Tyrobp(−/−) vs WT log_2_ fold-change of reactive astrocyte marker genes. in the transcriptome nominal p-value from the differential expression analysis is shown. ****p < 0.0001; ***p < 0.001; **p < 0.01; *p < 0.05

Subsequently, each of the WT and Q175 cohort samples (with or without *Tyrobp*) was acquired in DIA mode allowing for accurate and comprehensive quantification comparing the various genotypes. Overall, from this cohort we were able to identify 3,848 quantifiable protein groups with at least 2 unique peptides. Our approach allowed us to perform in-depth proteome analysis, and covered a dynamic range over 5 orders of magnitude by abundance level (Additional file 1: Fig. S7A). GO biological processes and cellular component correlated with overall protein abundances in the anticipated manner (Additional file 1: Fig. S7B). Our dataset included very well-defined striatal-enriched markers, such as PPP1R1B, PDE10A, ADCY5, and receptors, including DRD1, ADORA2A and GABRA1. All protein identifications and quantifications are included in Additional file 7: Table S6. Partial least squares-discriminant analysis revealed highly specific and separated clustering of all genotypes, with greater separation between WT and Q175 (Fig. 6A). We detected differentially expressed proteins in all comparisons examined (DEPs, FDR < 0.05 and absolute log_2_(fold change) > 0.2). We observed a higher number of DEPs in the Q175 vs WT comparison, in agreement with RNA-seq data, with 275 DEPs overlapping (Fig. 6B). Consensus path analysis revealed that downregulated proteins in Q175 striatum were enriched for GO terms related to the neuronal system (Fig. 6C and Additional file 8: Table S7). In agreement with transcriptomics data, we detected a small number of DEPs in the Q175-*Tyrobp*^*(−/−)*^ vs Q175 comparison, but importantly, these included a large reduction in C1Q and GFAP protein levels when *Tyrobp* was absent (Fig. 6D). Interestingly, we detected that deletion of *Tyrobp* in Q175 increases the levels of serine protease HTRA1, which is known to degrade aggregated and fibrillar tau [78]. In order to identify the most robust expression changes in Q175 and the impact of *Tyrobp* deletion in Q175 mice, we evaluated the correlation between gene and protein expression changes and examined the expression of those genes detected by both RNA-seq and proteomic profiling. Given the gap between numbers of significant DEGs and DEPs, we used a different threshold for each comparison. We used genes detected as DEGs with adjusted p-value < 0.05 in Q175 vs WT comparison, and genes detected with nominal p-value < 0.05 for Q175-*Tyrobp*^*(−/−)*^ vs Q175 comparison. Remarkably, we detected high concordant changes in the proteome and transcriptome in both cases (Fig. 6E, F). We observed that *Gfap* is the gene/protein with the highest fold-change. Importantly, we found that deletion of *Tyrobp* reduces both gene and protein expression of *C1q* (Fig. 7A) and *Gfap*. Using immunofluorescence, we obtained consistent experimental evidence to support the normalization of *Gfap* expression in Q175;*Tyrobp*^*(−/−)*^ mice (Fig. 7B). Further, we quantified aspects of astrocyte morphology in the four groups of mice and found a trend towards normalization of astrocytic branch number, process length, area of GFAP-positive cells, and soma area for the Q175;*Tyrobp*^*(−/−)*^ mice towards WT (Additional file 1: Fig. S8).

The lack of an increase in GFAP in the absence of TYROBP led us to specifically interrogate other astrocytic markers often associated with neurodegenerative disease and neuroinflammation, i.e., “neurotoxic reactive astrocytes” [79, 80]. Although the classification remains somewhat controversial and does not account for all astrocytic phenotypes, reactive astrocytes may be type A1, which are pro-inflammatory and neurotoxic, and A2, which are anti-inflammatory and neuroprotective [81, 82]. Microglia induce neurotoxic astrocytes via C1Q, TNFα, and IL-1α [81] and reduction in any one of these secreted molecules decreases astrocyte toxicity. We have already noted the decrease in *C1q* in Q175 mice on a *Tyrobp*-null background relative to Q175 alone. Consistent with the decrease in GFAP and immunocytochemically detected astrocytosis, there is a decrease in other pan-astrocytic markers (*Osmr* and *Serpina3*). Notably, additional A1 and A2 markers are also relatively decreased, suggesting that the progression of astrocytes through the various stages is halted (Fig. 7C).

**Fig. 8:**
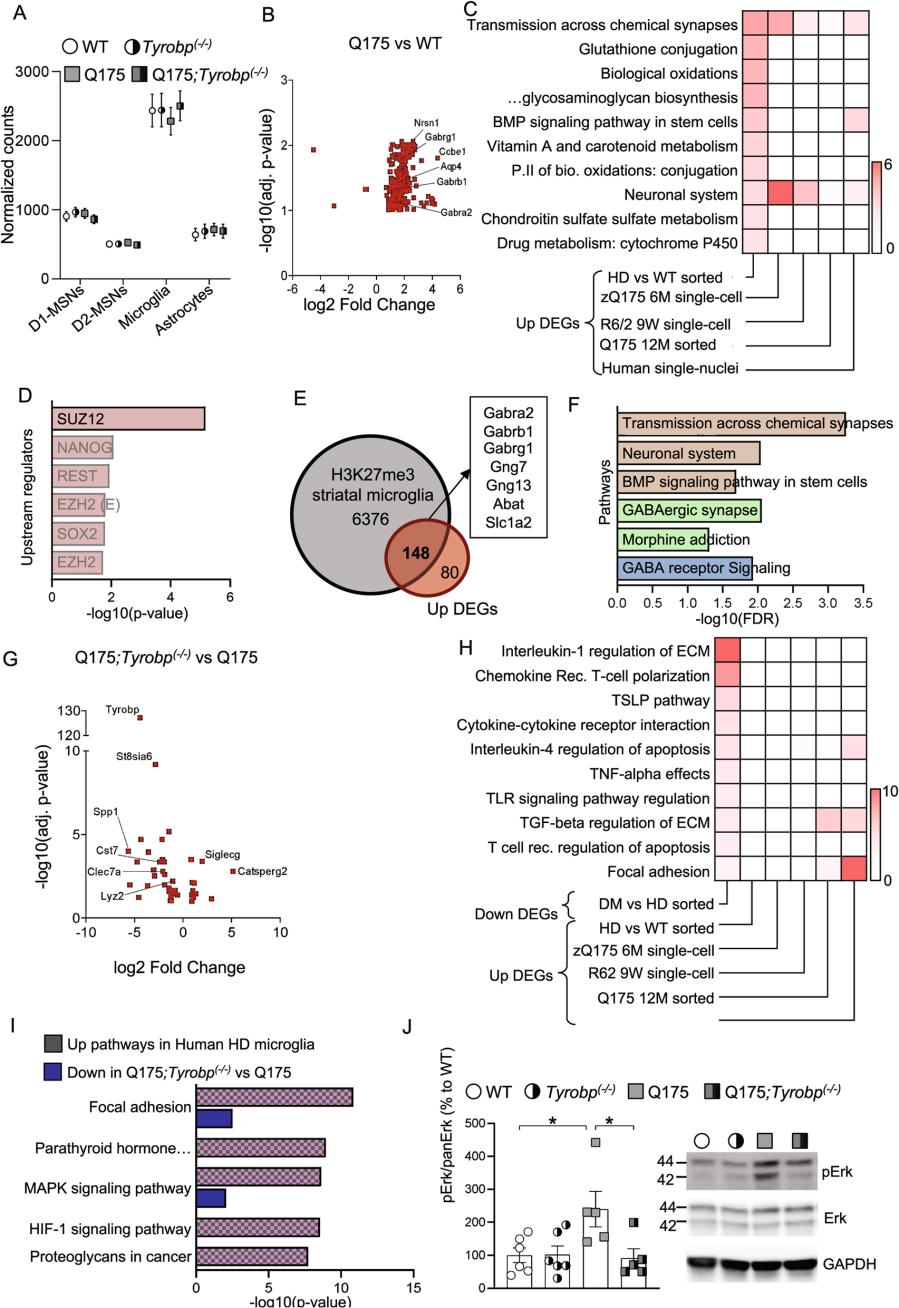
Analysis of the HD microglia-specific transcriptome and the consequences of *Tyrobp* deletion. RNA was purified from freshly isolated striatal microglia and then sequenced. **A** Analysis of global levels of cell type-specific transcripts from sorted mouse microglia RNA-seq samples using geometric mean of the normalized counts of cell type marker genes of Drd1 (direct)-MSNs, Drd2 (indirect) -MSNs, astrocytes and microglia. **B** Volcano plot illustrating the DEGs identified in Q175 vs WT comparison. Only the genes with FDR < 0.1 are shown. **C** Heatmap of binomial p-values for Bioplanet pathways upregulated in human and mouse datasets. **D** Predicted upstream regulators of upregulated genes from Q175 microglia. **E** Intersection between genes with H3K27me3 marks from striatal microglia and upregulated genes from Q175 microglia. **F** Pathways associated with upregulated DEGs with a H3K27me3 mark. Color denotes database (Brown: Bioplanet; Green: KEGG pathways; Blue: Wikpathways). **G** Volcano plot illustrating the DEGs identified in Q175;*Tyrobp*^(−/−)^ vs Q175 comparison. Only the genes with FDR < 0.1 are shown. **H** Heatmap of binomial p-values for Bioplanet pathways decreased in Q175;*Tyrobp*^(−/−)^, and integration with increased pathways from specified human and mouse datasets. **I** KEGG pathways enriched for upregulated genes in the microglial cluster of HD human snRNA-seq (pink/gray), integrated with the pathways downregulated by *Tyrobp* deletion in Q175 mice (blue). **J** Western blot and densitometric analysis of phosphorylated and total Erk protein in striatal samples. n = 5–6 mice per group. Quantitative analysis is shown as mean ± SEM. Each point represents data from an individual mouse. Statistical analysis was performed using Two-Way ANOVA. *p < 0.05

Cell-autonomous and non-cell-autonomous mechanisms may contribute to reactive microgliosis in HD brains. It has been previously demonstrated that mHtt expression in microglial cells is sufficient to elicit cell-autonomous transcriptional activation of pro-inflammatory genes *in vitro* [19]. However, we and others failed to detect activation of pro-inflammatory transcriptional pathways in HD mouse models. These data suggest that bulk transcriptomic analyses may not be sufficiently sensitive to detect microglial transcriptional alterations. To obtain a comprehensive and integrative picture of the transcriptional landscape of striatal HD microglia, and to evaluate its modulation by *Tyrobp* deletion, we isolated microglia from the striata of adult Q175 mice on a wild-type or homozygous *Tyrobp* KO background. Normalized counts from all samples were compared to striatal cell-type specific genes [83]. Microglial-specific genes are highly enriched in this data set compared with markers for D1- and D2-MSNs and astrocytes (Fig. 8A). There were a total of 228 differentially expressed genes in Q175 vs. WT microglia, and only 4 were downregulated (Fig. 8B; Additional file 9: Table S8). Surprisingly, pathway analysis revealed several families specifically associated with neuronal functions, including transmission across chemical synapses or neuronal systems (Fig. 8C). HD microglia show higher expression of GABA-related genes (i.e., *Gabra2*, *Gabrb1* and *Gabrg1*) and significant enrichment for glutathione conjugation and biological oxidations, pathways that include genes related to detoxification mechanisms, e.g. *Gstm1*, *Gsta4* and *Gstm5*.

To determine the reproducibility of the genes and pathways altered in mouse HD microglia, we performed pathway enrichment analysis for upregulated genes comparing control and HD microglial clusters from publicly available single-nuclei RNA-seq experiments. These sets include data from human caudate/putamen, and Q175 (6-month-old) and R6/2 (9-week-old) mouse models [84] and a recent study which profiled freshly isolated microglia from Q175 at 12 months of age [85]. We unexpectedly found that neuronal-related pathways are upregulated across all sets, including HD human microglia (Fig. 8C, Additional file 10: Table S9). Of note, pathways are shared despite the low overlap of specific genes across datasets. To identify potential key drivers of this transcriptional dysregulation, we performed ChEA analysis for upregulated genes. We observed that Suz12 is the only significant predicted upstream regulator of the up-regulated DEGs (Fig. 8D). Suz12 is a core component of the polycomb repressive complex 2 (PRC2) and is altered in HD [86]. PRC2 catalyzes Histone 3 Lysine 27 di- and trimethylation (H3K27me2 and H3K27me3), leading to gene repression [87]. For this reason, we intersected our upregulated DEGs with striatal microglia specific H3K27me3 marks [88], and found that 148 of the 228 up-regulated DEGs have a H3K27me3 histone mark in striatal microglia (Fig. 8E). Importantly, we observed that those genes are related to neuronal function (Fig. 8F). These data suggest that mHtt may disturb PRC2 function and thereby derepress the expression of neuronal genes in microglial cells. Whether the expression of neuronal genes in HD microglia contributes to HD pathophysiology is still unknown.

Next, we focused on how *Tyrobp* modulates the HD/Q175 microglia transcriptome. Comparing Q175;*Tyrobp*^*(−/−)*^ versus Q175 mice, we identified 43 DEGs (Fig. 8G), which primarily represented a decrease in the transcription of genes related to the *Tyrobp* network, including *Clec7a*, *Lyz2*, and *Spp1*. *Ccl3* and *Ccl4* were also identified, which were below the pAdj cutoff in the bulk sequencing. Notably, microglia homeostatic genes were not prominently dysregulated. We performed pathway enrichment analysis on the list of downregulated genes in Q175 *Tyrobp*^*(−/−)*^ versus Q175 mice (FDR < 0.1). Interleukin-1 regulation of extracellular matrix, Chemokine receptor T-cell polarization, TSLP pathway, Cytokine-cytokine receptor interaction and IL-4 regulation of apoptosis were the most significantly altered pathways in the Q175 *Tyrobp*^*(−/−)*^ versus Q175 mice comparison (Fig. 8H). As we previously detected in our bulk sequencing analysis, the pathways affected by *Tyrobp* deletion in Q175 mice are not activated in HD mouse models. However, IL-4 regulation of apoptosis, TGFβ regulation of extracellular matrix and focal adhesion are pathways downregulated by *Tyrobp* deletion in Q175 mice that are activated in human HD microglia (Fig. 8H; Additional file 11: Table S10). For this reason, we next focused on microglial-specific pathways activated in HD human brains (Fig. 8I). We observed that the top pathways enriched for upregulated DEGs detected in microglial cells from HD brains were shared with those pathways downregulated in Q175:*Tyrobp*^*(−/−)*^ when compared to Q175 (Fig. 8I). IL-2 signaling pathway and focal adhesion, two upregulated Bioplanet pathways in human HD microglia, were downregulated by *Tyrobp* deletion in HD mice (Fig. 8I, Additional file 11: Table S10).

We also noted that the MAPK signaling pathway is activated in human HD microglia and downregulated in Q175;*Tyrobp*^*(−/−)*^mice. Microglia-mediated pathogenic processes partly depend on upstream signaling events engaged by multiple pathological stimuli, probably including mHtt expression. Erk signaling regulates pro-inflammatory microglial activation in response to interferon γ [89, 90]. Also, it is an upstream regulator of several DAM genes and genetic risk factors for LOAD [91]. For this reason, we evaluated Erk phosphorylation status in the striatum of our mice. Although we did not perform this analysis in a cell-type specific manner, we observed that Erk is hyperphosphorylated in the striatum of HD models (Fig. 8J), as previously described [92–94]. Importantly, *Tyrobp* deletion in Q175 mice normalized phosphorylated Erk to wild-type levels (Fig. 8J). The Erk and pErk 42 and 44 kDa bands were both quantitated, and summed. These data demonstrate that *Tyrobp* is a candidate to not only normalize the pro-inflammatory pathways of human microglia, but also to restore, at least, Erk intracellular signaling pathways in mouse models.

## Discussion

In this study, we employed multiple approaches to identify *Tyrobp* as a mediator of microglial and neuronal dysfunction in HD. We demonstrated that constitutive deletion of endogenous *Tyrobp* in a mouse model of HD normalizes microglial morphology, impedes the reduction of PSD-95 and the increase in CD68, improves motor function, prevents astrogliosis, and downregulates transcriptomic pathways altered in HD human brain. We extended these observations by generating and integrating transcriptomic data from HD microglia and also observed important congruence between transcriptomics and proteomics in our study. We observed that *Tyrobp* deletion reduces the expression of genes belonging to pathways selectively activated in HD human microglia. Ultimately, our data lead to the proposed reduction of *Tyrobp* and/or its signaling pathway as a potential therapeutic approach to mitigate mHtt toxicity and promote neuronal function.

*TYROBP* was identified as a hub gene in a specific LOAD pro-inflammatory subnetwork [24]. Our group validated this observation in an APP/PSEN1 model of amyloidopathy and in a model of tauopathy, where complete deletion of *Tyrobp* is phenotypically beneficial [28–30]. In addition to AD, *TYROBP* was identified as a hub gene in a microglial module conserved between human aging and neurodegenerative diseases, including Parkinson's disease (PD) and HD [95]. HD and AD share some pathological hallmarks, including altered proteostasis and detrimental pro-inflammatory responses; however, the neuroinflammatory characteristics are different. HD brains show normal levels of immunoglobulins throughout the disease course, suggesting that there is no generalized activation of the adaptive immune response [12]. However, previous gene expression and network analyses restricted to HD samples did not identify a *TYROBP* network in HD with altered connectivity or expression [18, 96–98]. These previous studies, however, utilized microarray data. Here, we used the most recent cortical and striatal RNA-seq studies [31–33] to perform pathway analysis. RNA-seq has several benefits over microarrays, including higher resolution for the identification of low-abundance transcripts and the ability to distinguish expression profiles of closely related paralogues. Differential connectivity network analysis can capture more transcriptional information between cases and controls than was previously appreciated by differential expression alone. Here, we observed that “TYROBP causal network in microglia” is the most enriched pathway for the upregulated genes detected across all human HD RNA-seq datasets. Also, our PPI-network results showed that *TYROBP* is a potential hub gene in HD brains.

A caveat for some of our data is the issue regarding the interpretation of results from bulk RNA sequencing in neurodegenerative diseases in which significant neuronal death has already occurred, thereby skewing the percentage of cell subtypes within the entire population leading to potential artifact when determining enrichment. We recognize that this is a potentially confounding factor in our analysis of the *TYROBP* network in HD bulk seq databases, particularly human. This issue is particularly problematic if a gene is expressed in more than one cell type and moves in opposite directions depending on cell type, and also if there is a disparity between nuclear and cytoplasmic localization of a specific RNA species [99, 100]. That being said, brain TYROBP is expressed only in microglia and so enrichment of its network will arise from a single cell type. Unfortunately, we are limited by available databases and depth of sequencing, and so we are unable to reach a more definitive conclusion until data from single cell/nucleus sequencing of sufficient depth are available.

We demonstrate herein, as have many others, that HD mouse models accurately mimic human disease neuronal transcriptomic and proteomic alterations while other abnormalities, e.g., the activation of pro-inflammatory profiles, are not completely replicated. We observed that most of the inflammatory pathways transcriptionally increased or active in symptomatic and presymptomatic human caudate are downregulated by *Tyrobp* deletion in Q175 mice, despite the fact that they are not upregulated in Q175 only. We report that complement signature is downregulated in Q175;*Tyrobp*^*(−/−)*^ mice, which is in line with our previous findings in AD-related models. Although we did not detect activation of the complement pathway in HD mouse striatum, co-cultures of wild-type microglia with striatal neurons expressing mHtt displayed increased proliferation, elevated levels of cytokine IL-6 and complement components C1QA and C1QB, and take on a more amoeboid morphology [101]. These data point to a key role of *Tyrobp* in regulation of the complement system in yet another neurodegenerative disease. Importantly, postmortem studies of HD human tissue have identified increases in complement components, including C1Q, C3, C4, iC3b, and C9 [102]. Thus, in light of the PSD-95 and C1q findings, we suggest that microglial phagocytic activity is reduced in HD mice in the absence of *Tyrobp*, leading to a preservation of synapses. This would be consistent with a recent report specifically studying the role of C1Q and synapse preservation in HD [61].

We also generated an unbiased transcriptomic dataset of freshly isolated microglia from Q175 striatum, with and without *Tyrobp*, where we observed similar effects of *Tyrobp* deletion as in AD models. In the presence of *Tyrobp*, we detected increased expression of genes related to pro-inflammatory processes such as *Pla2g5*, *Trcp6* or *Pdpn*. These genes have been linked to the regulation of inflammatory molecule synthesis and microglial mobility and phagocytosis [103–105]. We also report activation of glutathione conjugation genes, which may reflect a compensatory mechanism to enhance detoxification mechanisms, but also could be part of a pathological mechanism. For example, *Gstm1*, which is upregulated in HD microglia, is a glutathione S-transferase that contributes to astrocyte-mediated enhancement of microglia activation during brain inflammation [106]. Nonetheless, the overall transcriptome did not reflect immune activation.

In the absence of *Tyrobp*, however, the down-regulated genes present in the microglial transcriptome offer mechanistic pathways for how *Tyrobp* deletion leads to prevention of aspects of the HD phenotype. In this regard, we want to emphasize that the beneficial effects of down-regulation of these pathology-related genes appears to be beneficial even though they are at homeostatic levels in Q175 alone, i.e., not elevated. It is a novel concept that down-regulation of this group of microglial genes from homeostatic levels can be beneficial in neurodegenerative disease. We have already discussed the ramifications of a decrease in C1Q, which include protection from abnormal synapse engulfment and also prevention of toxic astrogliosis, although the A1 astrocyte gene signature is not apparent in Q175 mice despite its presence in human HD  [81, 99]. It has already been reported that synaptic engulfment is decreased in an HD model following reduction in C1Q [66]. Aberrant synaptic engulfment is likely further diminished by the decrease in *Spp1* expression, as SPP1 has been shown to be required for aberrant synaptic engulfment in AD [107], thus potentially acting synergistically with the decrease in C1Q. The decrease in IGF-1 is of interest, since higher levels have been associated with lower cognitive scores in HD [108]. Finally, most recently, it was reported that *Cll* gene family interactions with their neuronal receptor CCR5 increases autophagy in HD which is relieved by a decrease in CCR5 [109]. We hypothesize that a decrease in the chemokine ligands would potentially have the same effect as does down-regulation of the receptor. A decrease in KLK8 may synergize with the decrease in autophagy and also in promotion of synaptic plasticity, as it does in an AD model [110].

There is expression of GABA receptors in microglia [111–113], which are essential for inhibitory connectivity. Removal of microglial GABA receptors alters inhibitory connectivity, induces behavioral abnormalities in mice and, importantly, causes a downregulation of synaptic pruning-related genes [111]. Overexpression of neuronal-related genes in HD microglia is described in other HD models and also in single-nuclei from HD human cortex. A potential mechanism is that perhaps mHtt disrupts the PRC2 complex, causing a de-repression of neuronal genes in microglia. Although the consequences of GABA receptor overexpression in microglia are not known, one plausible consideration is that it is a compensatory mechanism to attenuate mHtt-induced pro-inflammatory responses. In vitro, GABA reduces LPS-induced microglial IFN-γ, IL-6 and TNFα production [113] and in vivo, activation of GABA B receptors after facial nerve axotomy strongly decreases LPS-induced secretion of certain cytokines, including IL-6 and IL-12p40 [112]. These data suggest that HD microglia may overexpress GABA receptors in an attempt to reduce striatal inflammation as one of several potentially compensatory mechanisms activated in the striatum of HD models [114]. We also detected overexpression of *Fbxo2* and *Scrg1* in HD microglia. *Fbxo2* mediates clearance of damaged lysosomes and modifies neurodegeneration in Nieman-Pick C models [115], suggesting that HD microglia may be attempting to improve lysophagy pathways. *Scrg1* suppresses LPS‑induced Ccl22 production in monocyte/macrophage-like cells [116].

## Conclusions

In summary, we demonstrate that although microglia from HD mouse models do not fully recapitulate the human HD microglia transcriptome, deletion of *Tyrobp* in a full-length HD mouse model corrects potentially pathogenic pathways activated in HD microglia. Therefore, *Tyrobp* deletion improves the behavior of APP/PSEN1, MAPT^P301S^ and Q175 mice, in each case, decreasing many of the same genes even if they are not pathologically increased in the presence of *Tyropb*. Our focus was on the striatum, an area not assayed in the AD studies. This is the first instance of abrogation of abnormal motor behavior following *Tyrobp* manipulation. Importantly, the microglial sequencing was performed in microglia isolated from the striatum. Although *Tyrobp* is expressed ubiquitously in microglia, there is much regional heterogeneity in microglial phenotype, and it was not known in advance how striatal microglia would respond to *Tyrobp* deletion. Notably, the effect of *Tyrobp* deletion on astrogliosis is not seen in AD models, and this very specifically is seen in the striatum in this report. Finally, the *Tyrobp*/AD model work involves mice in which endogenous *Tyrobp* expression increases with disease stage, whereas it is not elevated at this age in the HD model, and so this is the first report showing that a decrease in microglial activity from homeostatic levels by *Tyrobp* deletion has definitive impact on a disease phenotype. Importantly, deletion of *Tyrobp* has very specific cell-autonomous effects on the microglial inflammation-related transcriptome, without restoration of the transcriptome in the MSNs, implying that at this stage of disease, motor function can be improved without amelioration of neuronal pathology. These data imply that downregulation of a *Tyrobp* network in the presence of a proteinopathy can be beneficial even if not pathologically elevated, regardless of baseline levels and MSN transcriptional dysregulation. Further studies will be performed with a conditional deletion of *Tyrobp* to better mimic what might be attempted with therapeutic manipulation of this pathway.

### Supplementary Information

**Additional file 1: Fig. S1.** (A) Schematic of the biochemical and behavioral studies on WT and Q175 mice with and without *Tyrobp*. (B) RT-qPCR of Aif1 (Iba1 gene) mRNA (left) and WB of Iba1 protein (right) in the striatum of WT and Q175 mice with and without *Tyrobp* (10 months of age), n = 6 mice per group. Each point represents data from an individual mouse. **Fig. S2.** (A) Representative immunofluorescent images of of CD68 cells colabeled with Iba1. Virtually all cells were double-labeled in all genotypes. (B) Quantification of the CD68+ cells were positive for Iba1+ cells. Averaged number of CD68+ cells were positive for Iba1+ cells from three fields from 3 different striatal sections is represented per group as mean ± SEM (WT n= 3; Q175 n = 3; *Tyrobp*(-/-) n= 3 Q175;*Tyrobp*(-/-) n = 3). Each point represents data from an individual mouse. Scale bar = 5 μm. **Fig. S3.** Number of mHtt+ particles was evaluated in the dorsal striatum of Q175 mice at 9 months of age. Averaged number of mHtt+ particles from four fields from 3 different striatal sections is represented per group as mean ± SEM (Q175 n = 6; Q175;*Tyrobp*(-/-) n = 6). Each point represents data from an individual mouse. Scale bar = 50 μm. **Fig. S4.** Cognitive behavior was evaluated in Q175 mice at 9 months of age using the Novel Object Recognition test (NOR). Time exploring and percentage exploring each arm is represented per group as mean ± SEM (WT n = 7; *Tyrobp*(-/-) n = 6; Q175 n = 13; Q175;*Tyrobp*(-/-) = 14). Each point represents data from an individual mouse. Statistical analysis was performed unpaired t-test comparing old vs new arm. *p < 0.05; ***p < 0.001. **Fig. S5.** (A) RT-qPCR, (B) WB and (C) immunofluorescence analysis of DARPP-32 in the striatum of WT and Q175 mice with and without *Tyrobp* (10 months of age), n = 6 mice per group. Data represent the mean ± SEM. Each point represents data from an individual mouse. Two-way ANOVA followed by Bonferroni’s post hoc test, *p < 0.05; **p < 0.01; ***p < 0.001. Scale bar = 200 μm. **Fig. S6.** Normalized counts of striatal-specific genes detected in the striatum of WT and Q175 mice with and without *Tyrobp* (10 months of age) by bulk RNAseq, n = 6 mice per group. **Fig. S7.** (A) Ranking of brain proteins by normalized protein abundance from highest to lowest. (B) Top enrichment for each quartile is displayed for GO categories “biological process” and “cellular component”; FDR, Benjamini-Hochberg-corrected false discovery rate. OR, Odds Ratio. **Fig. S8.** Quantification of the morphology of GFAP cells in the striatum of WT and Q175 mice with and without *Tyrobp*.

**Additional file 2: Table S1.** Pathway enrichment analysis for upregulated genes detected in human HD datasets. Top 10 significant pathways are shown (WikiPathway 2021 Human).

**Additional file 3: Table S2.** Overrepresented Gene Ontology (GO) terms within the STRING protein-protein interaction (PPI) network.

**Additional file 4: Table S3.** DEGs detected in bulk transcriptomics. Q175 vs WT comparison.

**Additional file 5: Table S4.** GO terms from GSEA analysis performed in Q175 vs WT comparison.

**Additional file 6: Table S5.** KEGG pathways from GSEA analysis performed by Agus *et al*., 2019 in symptomatic HD vs control BA9 samples.

**Additional file 7: Table S6.** All quantifiable protein groups.

**Additional file 8: Table S7.** Consensus path analysis on proteomic data.

**Additional file 9: Table S8.** Differentially expressed genes detected in Q175 vs WT sorted microglia.

**Additional file 10: Table S9.** Pathway enrichment analysis for microglial datasets.

**Additional file 11: Table S10.** Pathway enrichment analysis for microglial datasets integrated with downregulated pathways detected in Q175 vs Q175;*Tyrobp*(-/-).

**Additional file 12: Table S11.** Isolation scheme of the DIA method.

## Data Availability

Raw data and processed information of the RNA-seq experiments generated in this article were deposited at the Gene Expression Omnibus repository under the accession numbers GSE193573 (bulk RNA-seq) and GSE195633 (microglia RNA-seq). Raw data and complete MS data sets have been uploaded to the Center for Computational Mass Spectrometry, to the MassIVE repository at UCSD, and can be downloaded using the following link: ftp://massive.ucsd.edu/v07/MSV000088643/, https://massive.ucsd.edu/ProteoSAFe/dataset.jsp?task=2194e8c2d8cd479780e75e917f946e2d (MassIVE ID number: MSV000088643; ProteomeXchange ID: PXD030747).

## Abbreviations

AD: Alzheimer’s disease
DAM: Disease-associated microglia
DEGs: Differentially expressed genes
DEPs: Differentially expressed proteins
DIA: Data-independent acquisition
Erk: Extracellular signal-regulated protein kinase
FDR: False discovery rate
H3K27me2: Histone 3 Lysine 27 dimethylation
H3K27me3: Histone 3 Lysine 27 trimethylation
Gfap: Glial fibrillary acidic protein
GSEA: Gene set enrichment analysis
HD: Huntington's disease
HTT: Huntingtin human gene
Htt: Huntingtin mouse protein
Iba1: Ionized calcium binding adaptor molecule 1
IL-: Interleukin-
LOAD: Late onset Alzheimer’s disease
MCODE: Molecular complex detection
mHtt: Mutant huntingtin
MSNs: Medium spiny neurons
PBS: Phosphate-buffered saline
PD: Parkinson's disease
PPI: Protein–protein interaction
PRC2: Polycomb repressive complex 2
PSD-95: Post-synaptic density protein of 95kDa
qPCR: Quantitative polymerase chain reaction
RNA-seq: Ribonucleic acid sequencing
rcf: Relative centifugal force
SEM: Standard error of the mean
TBS: Tris-buffered saline
Tyrobp: TYRO protein tyrosine kinase-binding protein
WT: Wild-type
