# Supplementary material for: TYROBP/DAP12 knockout in Huntington’s disease Q175 mice cell-autonomously decreases microglial expression of disease-associated genes and non-cell-autonomously mitigates astrogliosis and motor deterioration
Source: J Neuroinflammation. 2024 Mar 8;21:66. doi: 10.1186/s12974-024-03052-4 (PMC10924371; doi:10.1186/s12974-024-03052-4)
Supplement: Supplementary file 1 — Additional file 1: Fig. S1. (A) Schematic of the biochemical and behavioral studies on WT and Q175 mice with and without Tyrobp. (B) RT-qPCR of Aif1 (Iba1 gene) mRNA (left) and WB of Iba1 protein (right) in the striatum of WT and Q175 mice with and without Tyrobp (10 months of age), n = 6 mice per group. Each point represents data from an individual mouse. Fig. S2. (A) Representative immunofluorescent images of of CD68 cells colabeled with Iba1. Virtually all cells were double-labeled in all genotypes. (B) Quantification of the CD68+ cells were positive for Iba1+ cells. Averaged number of CD68+ cells were positive for Iba1+ cells from three fields from 3 different striatal sections is represented per group as mean ± SEM (WT n= 3; Q175 n = 3; Tyrobp(-/-) n= 3 Q175;Tyrobp(-/-) n = 3). Each point represents data from an individual mouse. Scale bar = 5 μm. Fig. S3. Number of mHtt+ particles was evaluated in the dorsal striatum of Q175 mice at 9 months of age. Averaged number of mHtt+ particles from four fields from 3 different striatal sections is represented per group as mean ± SEM (Q175 n = 6; Q175;Tyrobp(-/-) n = 6). Each point represents data from an individual mouse. Scale bar = 50 μm. Fig. S4. Cognitive behavior was evaluated in Q175 mice at 9 months of age using the Novel Object Recognition test (NOR). Time exploring and percentage exploring each arm is represented per group as mean ± SEM (WT n = 7; Tyrobp(-/-) n = 6; Q175 n = 13; Q175;Tyrobp(-/-) = 14). Each point represents data from an individual mouse. Statistical analysis was performed unpaired t-test comparing old vs new arm. *p < 0.05; ***p < 0.001. Fig. S5. (A) RT-qPCR, (B) WB and (C) immunofluorescence analysis of DARPP-32 in the striatum of WT and Q175 mice with and without Tyrobp (10 months of age), n = 6 mice per group. Data represent the mean ± SEM. Each point represents data from an individual mouse. Two-way ANOVA followed by Bonferroni’s post hoc test, *p < 0.05; **p < 0.01; ***p < 0.001. Scale bar [file 12974_2024_3052_MOESM1_ESM.pdf]

A

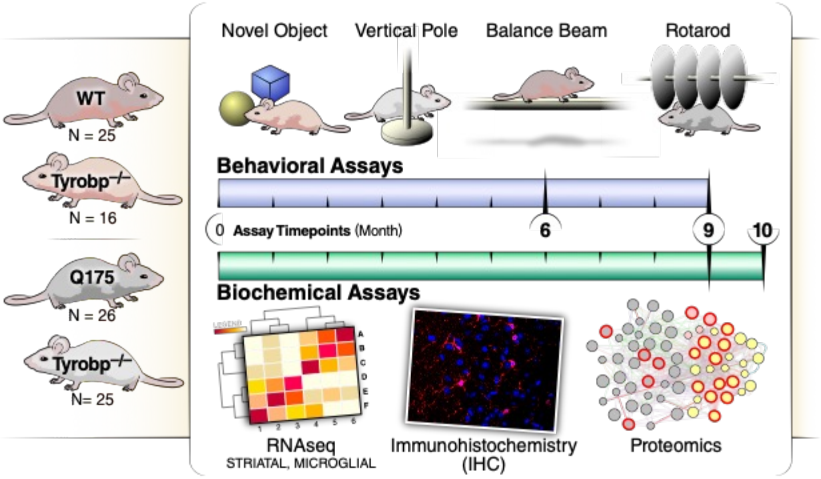

B

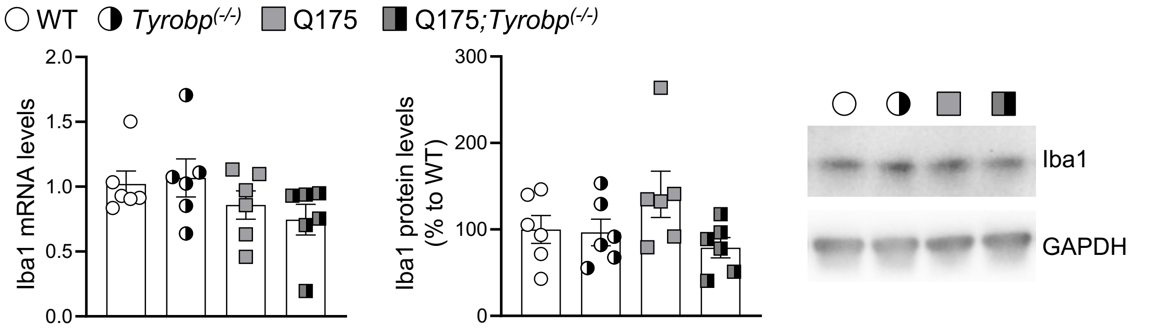

Supplementary Fig. 1. (A) Schematic of the biochemical and behavioral studies on WT and Q175 mice with and without *Tyrobp*. (B) RT-qPCR of *Aif1* (Iba1 gene) mRNA (left) and WB of Iba1 protein (right) in the striatum of WT and Q175 mice with and without *Tyrobp* (10 months of age), n = 6 mice per group. Each point represents data from an individual mouse.

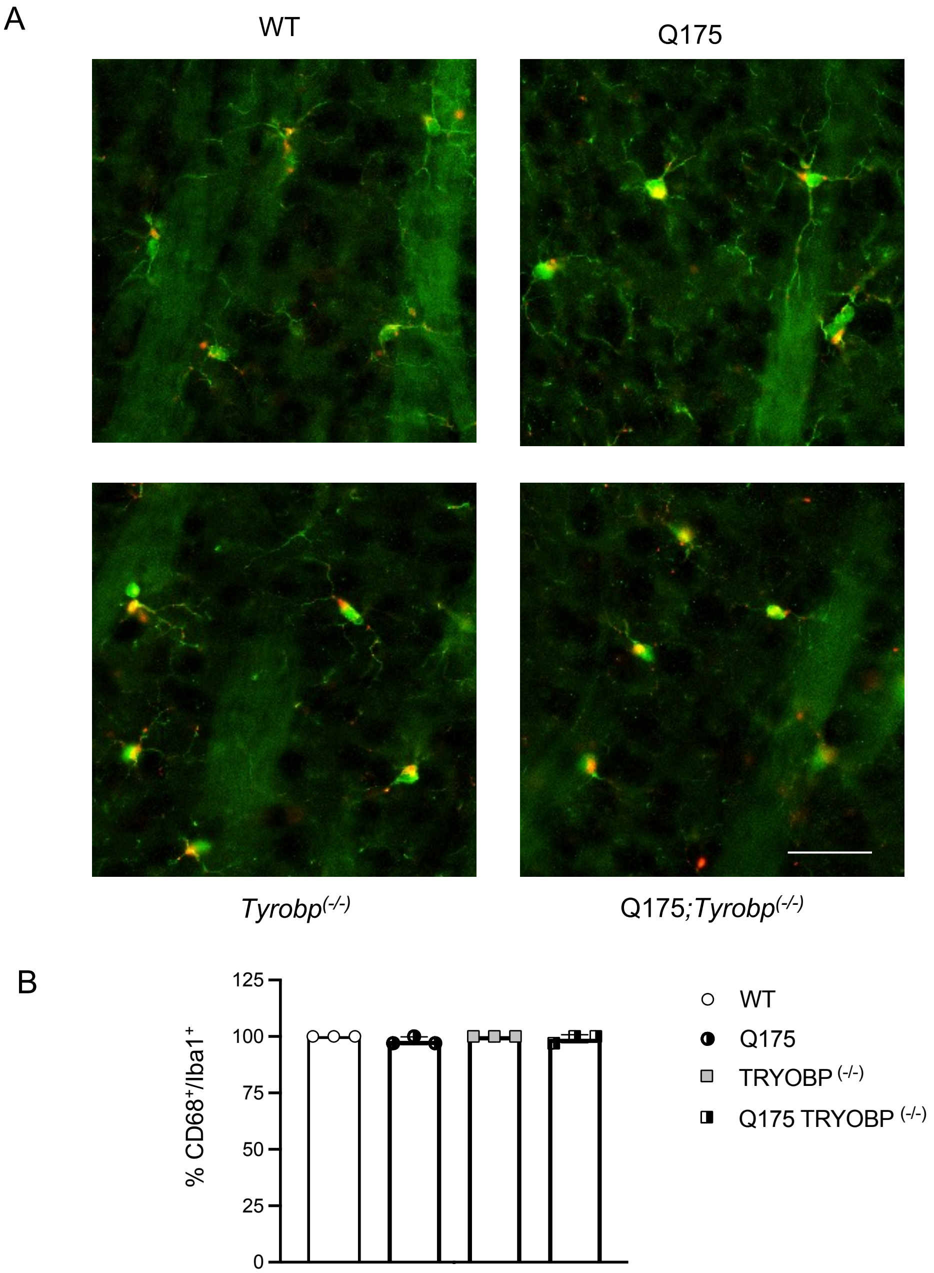

Supplementary Fig. 2. (A) Representative immunofluorescent images of of CD68 cells colabeled with Iba1. Virtually all cells were double-labeled in all genotypes. (B) Quantification of the $CD68^{+}$ cells were positive for $Iba1^{+}$ cells. Averaged number of $CD68^{+}$ cells were positive for $Iba1^{+}$ cells from three fields from 3 different striatal sections is represented per group as mean ± SEM (WT n= 3; Q175 n = 3; $Tyrobp^{(-/-)}$ n= 3 Q175;$Tyrobp^{(-/-)}$ n = 3). Each point represents data from an individual mouse. Scale bar = 5 μm.

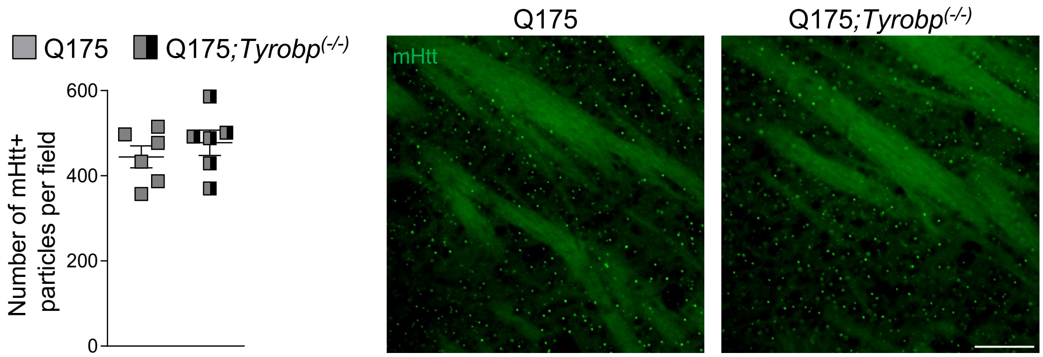

Supplementary Fig. 3. Number of mHtt+ particles was evaluated in the dorsal striatum of Q175 mice at 9 months of age. Averaged number of mHtt+ particles from four fields from 3 different striatal sections is represented per group as mean ± SEM (Q175 n = 6; Q175;Tyrobp(-/-) n = 6). Each point represents data from an individual mouse. Scale bar = 50 µm.

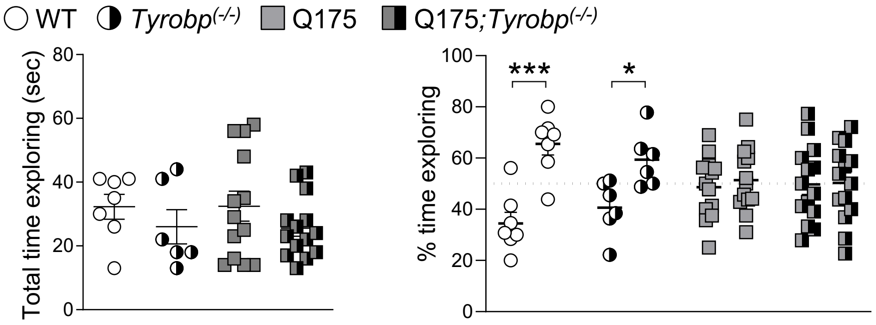

Supplementary Fig. 4. Cognitive behavior was evaluated in Q175 mice at 9 months of age using the Novel Object Recognition test (NOR). Time exploring and percentage exploring each arm is represented per group as mean ± SEM (WT n = 7; Tyrobp(-/-) n = 6; Q175 n = 13; Q175;Tyrobp(-/-) = 14). Each point represents data from an individual mouse. Statistical analysis was performed unpaired t-test comparing old vs new arm. *p < 0.05; ***p < 0.001.

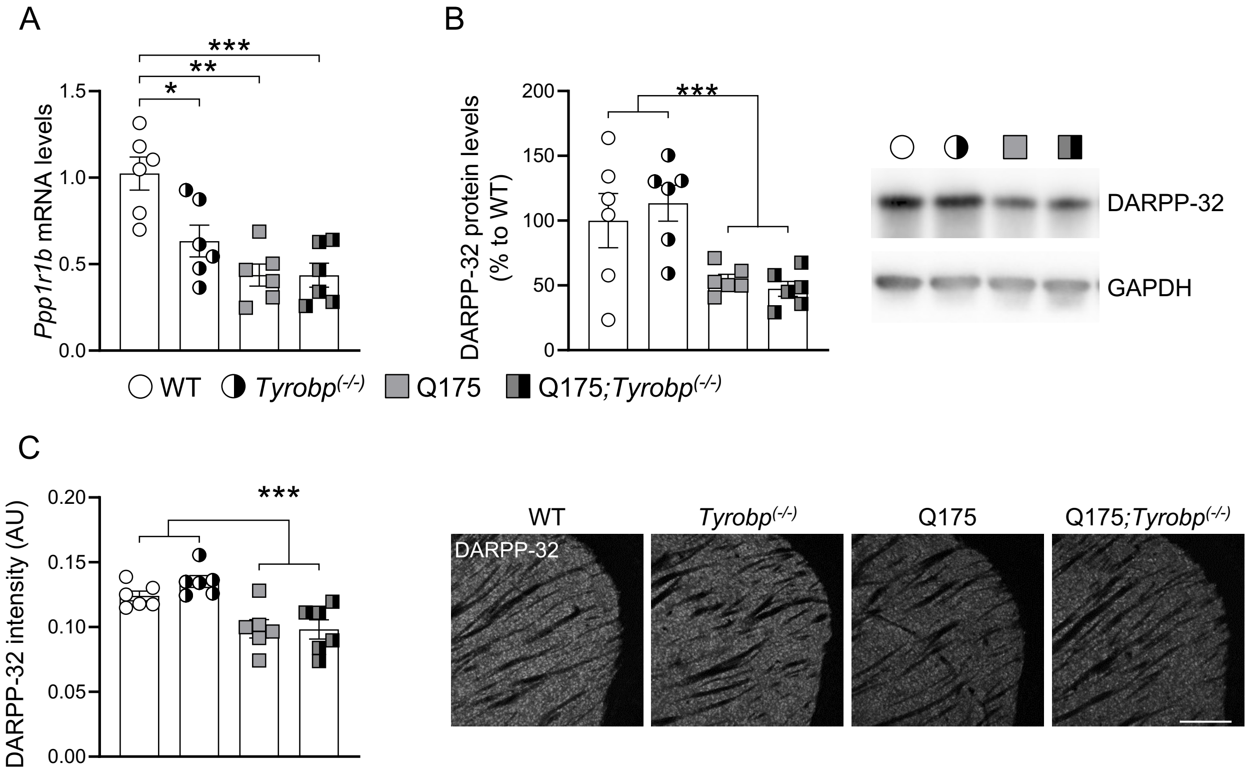

Supplementary Fig. 5. (A) RT-qPCR, (B) WB and (C) immunofluorescence analysis of DARPP-32 in the striatum of WT and Q175 mice with and without *Tyrobp* (10 months of age), n = 6 mice per group. Data represent the mean ± SEM. Each point represents data from an individual mouse. Two-way ANOVA followed by Bonferroni's post hoc test, *p < 0.05; **p < 0.01; ***p < 0.001. Scale bar = 200 μm.

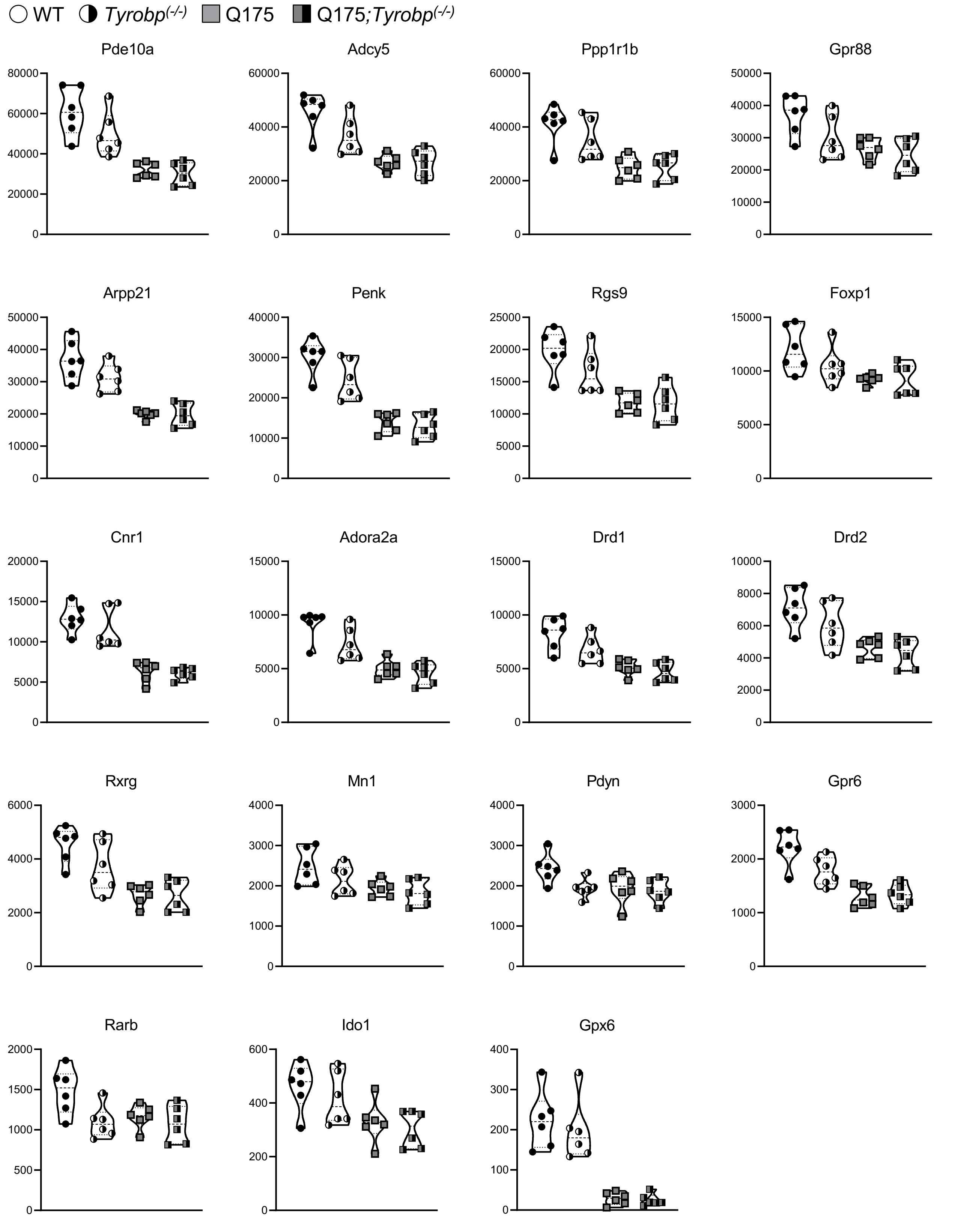

Supplementary Fig. 6. Normalized counts of striatal-specific genes detected in the striatum of WT and Q175 mice with and without Tyrobp (10 months of age) by bulk RNAseq, n = 6 mice per group.

A

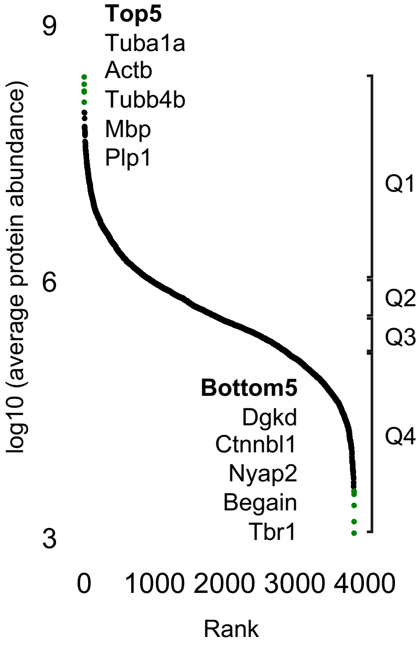

B

Quartile	GO Biological Process 2021	FDR	OR
Q1:	aerobic electron transport chain	1.403E-35	33.1
Q2:	cytoplasmic translation	1.677E-22	14.26
Q3:	post-Golgi vesicle-mediated transport	4.992E-07	6.63
Q4:	mitochondrial translational elongation	2.689E-10	7.94

Quartile	GO Cellular Component 2021	FDR	OR
Q1:	focal adhesion	2.217E-51	8.67
Q2:	cytosolic large ribosomal subunit	5.144E-14	14.61
Q3:	late endosome	3.342E-07	3.98
Q4:	mitochondrial inner membrane	0.0001579	2.67

Supplementary Fig. 7. (A) Ranking of brain proteins by normalized protein abundance from highest to lowest. (B) Top enrichment for each quartile is displayed for GO categories "biological process" and "cellular component"; FDR, Benjamini-Hochberg-corrected false discovery rate. OR, Odds Ratio.

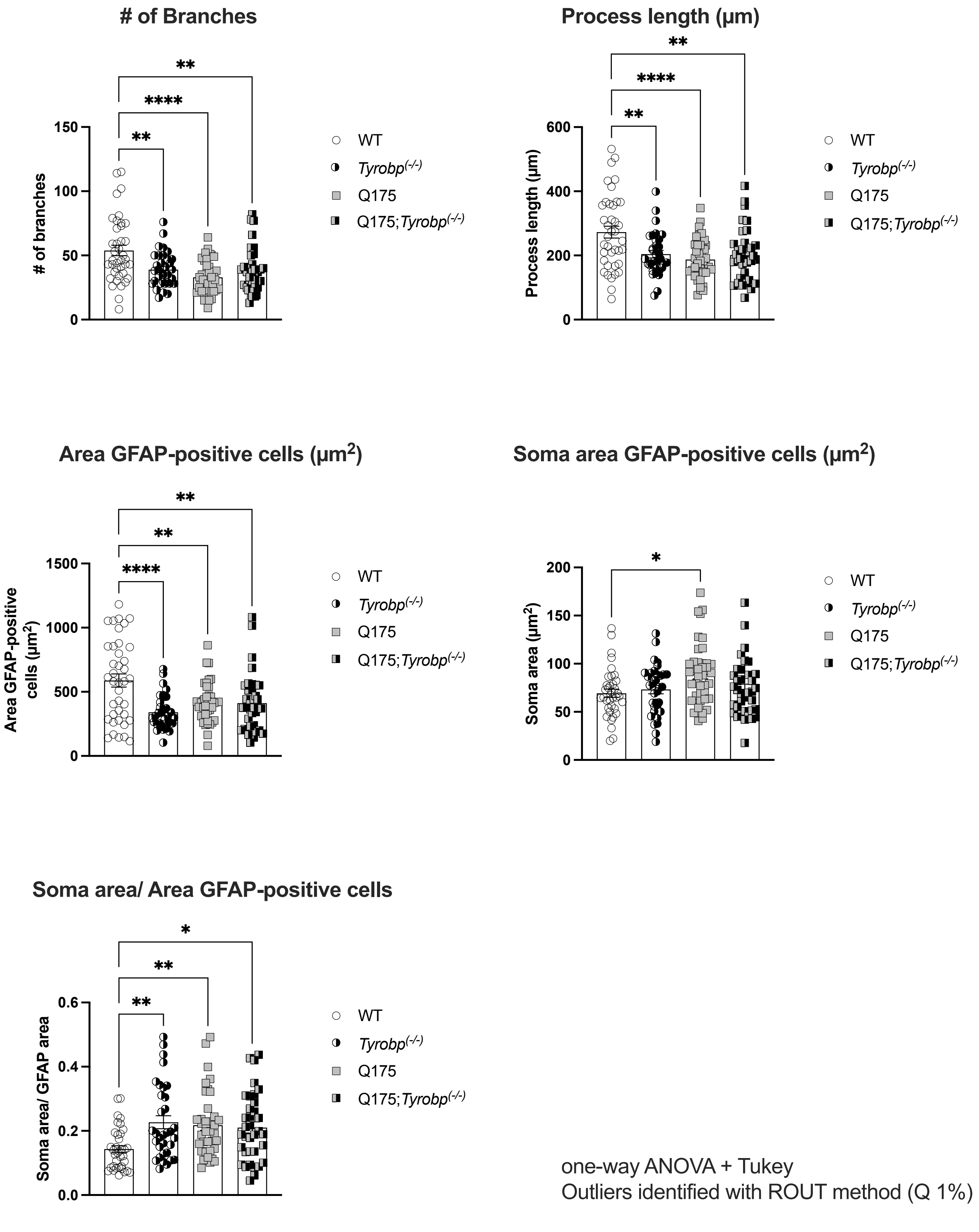

Supplementary Fig. 8. Quantification of the morphology of GFAP cells in the striatum of WT and Q175 mice with and without *Tyrobp*.
